# Nanosecond-scale single-molecule reaction dynamics for scalable synthesis on a chip

**DOI:** 10.1093/nsr/nwaf172

**Published:** 2025-04-26

**Authors:** Chen Yang, Shuyao Zhou, Yilin Guo, Zexi Hou, Junhao Li, Zhirong Liu, Zitong Liu, Deqing Zhang, Yanwei Li, Kendall N Houk, Xuefeng Guo

**Affiliations:** Beijing National Laboratory for Molecular Sciences, National Biomedical Imaging Center, College of Chemistry and Molecular Engineering, Peking University, Beijing 100871, China; Beijing National Laboratory for Molecular Sciences, National Biomedical Imaging Center, College of Chemistry and Molecular Engineering, Peking University, Beijing 100871, China; Beijing National Laboratory for Molecular Sciences, National Biomedical Imaging Center, College of Chemistry and Molecular Engineering, Peking University, Beijing 100871, China; Environment Research Institute, Shandong University, Qingdao 266237, China; Beijing National Laboratory for Molecular Sciences, National Biomedical Imaging Center, College of Chemistry and Molecular Engineering, Peking University, Beijing 100871, China; Beijing National Laboratory for Molecular Sciences, National Biomedical Imaging Center, College of Chemistry and Molecular Engineering, Peking University, Beijing 100871, China; State Key Laboratory of Applied Organic Chemistry, College of Chemistry and Chemical Engineering, Lanzhou University, Lanzhou 730000, China; Beijing National Laboratory for Molecular Sciences, CAS Key Laboratory of Organic Solids, Institute of Chemistry, Chinese Academy of Sciences, Beijing 100190, China; Environment Research Institute, Shandong University, Qingdao 266237, China; Department of Chemistry and Biochemistry, University of California, Los Angeles, Los Angeles, CA 90095, USA; Beijing National Laboratory for Molecular Sciences, National Biomedical Imaging Center, College of Chemistry and Molecular Engineering, Peking University, Beijing 100871, China; Center of Single-Molecule Sciences, Frontiers Science Center for New Organic Matter, College of Electronic Information and Optical Engineering, Nankai University, Tianjin 300350, China

**Keywords:** reaction mechanism, electrostatic catalysis, molecular electronics, nanosecond resolution measurement, on-chip synthesis

## Abstract

Reaction mechanism studies typically involve the characterization of products, and intermediates are often characterized by (sub)millisecond techniques, such as nuclear magnetic resonance, while femto/attosecond spectroscopies are used to elucidate the evolution of transition states and electron dynamics. However, due to the lack of detection techniques in the microsecond to nanosecond range, as well as the emergent complexity with increasing scale, most of the proposed intermediates have not yet been detected, which significantly hinders reaction optimization. Here, we present such a nanosecond-scale real-time single-molecule electrical monitoring technique. Using this technique, a series of hidden intermediates in an example Morita-Baylis-Hillman reaction were directly observed, allowing the visualization of the reaction pathways, clarification of the two proposed proton transfer pathways, and quantitative description of their contributions to the turnover. Moreover, the emergent complexity of the catalysis, including the catalysis oscillation effect and the proton quantum tunneling effect, is further unveiled. Finally, this useful yet low-yield reaction was successfully catalyzed by the application of an electric field, leading to a high turnover frequency (∼5000 s^−1^ at a 1 V bias voltage). This new paradigm of mechanistic study and reaction optimization shows potential application in scalable synthesis by integrated single-molecule electronic devices on chip.

## Teaser

Real-time single-molecule electrical monitoring at the nanosecond scale reveals hidden reaction steps in catalytic processes, enabling efficient on-chip chemical synthesis through electrostatic catalysis.

## INTRODUCTION

A comprehensive understanding of mechanistic features is important for optimizing chemical reactions. For a complex system, the elucidation of the mechanism ideally involves isolable intermediates to delineate the reaction pathway. However, most of these intermediates exist in the range between sub-microseconds [1–3] and tens of femtoseconds [4,5]. Emergent complexity, such as interactions or interferences among the elementary steps and catalysis cycles, should also be considered [6]. Single-molecule studies with high temporal resolution can provide a new way to study the mechanism [7].

**Figure 1.**
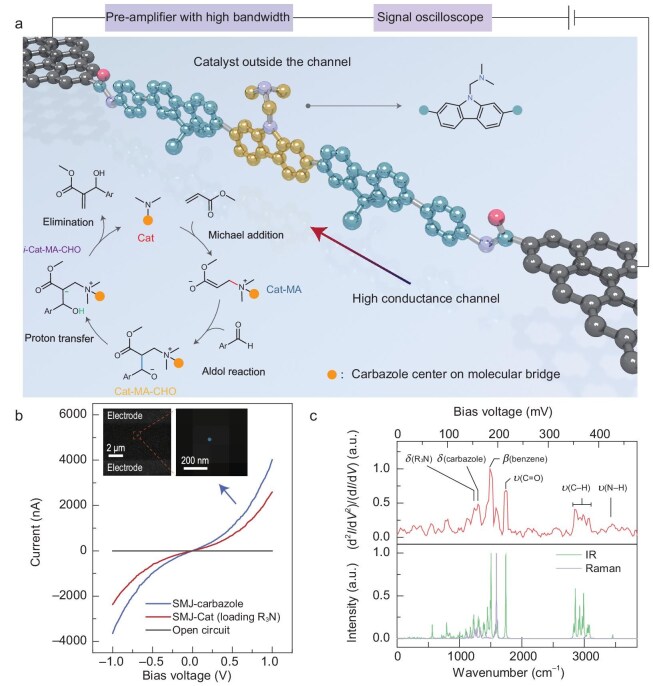
Preparation and characterization of a single-catalyst device. (a) Schematic diagram of a single-catalyst device, showing electrical monitoring with nanosecond resolution. The bottom left inset shows the proposed MBH reaction mechanism. (b) Scanned *I*–*V* curves before molecular connection (gray), after integration of the carbazole-centered molecular bridge (violet), and after further loading of the R_3_N catalytic center (red). After anchoring the carbazole molecule, the single-molecule connection was characterized by electroluminescence using a 4 V applied bias voltage. The stochastic optical reconstruction indicates only one molecule connection as shown in the two insets. (c) After loading the R_3_N catalytic center, the IETS of the molecule was characterized at 2 K using an AC modulation of 21.2 mV at a frequency of 661 Hz. Bottom: simulated infrared and Raman spectra of the corresponding molecular bridge. The peaks assigned to specific vibrational modes are marked in the IETS (*V* = *hω*/*e*). The characteristic peaks of *δ* (carbazole) (∼160 mV), *ν* (C–H) (350∼380 mV) and *δ* (R_3_N) (∼154 mV) were detected. In addition, the specific peaks of *ν* (C=O) and *ν* (N–H) in the amide bond were detected at ∼213 mV and ∼425 mV, respectively.

An attractive example to demonstrate the power of single-molecule investigations is the Morita-Baylis-Hillman (MBH) reaction, which has become a touchstone for mechanism studies [8,9]. This reaction constructs a C–C bond accompanied by multiple functional groups (Fig. 1a), meeting the requirements of atom economy and chemical selectivity, and it is therefore widely used for organic synthesis [10,11]. The proposed mechanism includes Michael addition, aldol reaction and subsequent proton transfer to enable the final elimination [9] (Fig. 1a, bottom left). However, the vast majority of intermediates during the catalysis remain undetected owing to the complex energy profile and numerous zwitterionic intermediates [8,9]. The mechanism of proton transfer remains controversial. A concerted proton shuttle mechanism mediated by a protonic solvent molecule is common, especially in life processes [9,12]. Singleton's studies, including solvent kinetic isotope effects, support the stepwise acid-base process [8], but pathways have not been directly observed. Given the complex process, slow reaction rates in some substrate cases [13–15], and the errors in computational simulations [8,16], understanding the mechanism has been very challenging.

Single-molecule detection, particularly electrical detection [17,18], focuses on the molecular conductance, which reflects the chemical structure [19] and conformation [20] of the molecule during the reaction. However, complex solution conditions and fast reaction dynamics pose formidable challenges for MBH reaction characterization. Using graphene as point electrodes to anchor a single molecule by two covalent bonds provides a determined interface coupling (leading to a narrow dynamic range of current fluctuation) and high tolerance to solution environments [21]. Furthermore, a one-molecule set-up enables real-time monitoring of the reaction rates, shedding light on the inherent reaction mechanism. However, considering the weak signal of a single molecule, accurate detection requires high (logarithmic) amplification [22] (usually 10^6^–10^9^) and relative long-interval integration (usually, MHz ∼ kHz pass bandwidth), sacrificing time resolution. Currently, state-of-the-art single-molecule electrical detection approaches ∼μs-scale time resolution based on linear amplification [19,23]. Therefore, fast dynamics with ∼ns-scale lifetime intermediates require further development of the time resolution, including improvements in the electrical signal amplitude, corresponding pass bandwidth and sampling rate.

Here, we report a universal strategy capable of describing the fast dynamics, using the MBH reaction as a case study. A highly conjugated molecular bridge was integrated into graphene electrodes via covalent bonds, meeting the requirement for strong electrical signals. The catalyst center was loaded onto the carbazole unit (yellow) of the molecular bridge, weakening the coupling with the electrodes by several spacer groups (blue) (Fig. 1a). The obtained electrical signal was amplified by a cascade, ensuring a bandwidth of 200 MHz, as close as possible to the resistor–capacitor limit of the circuit itself. Finally, we used a high-sampling-rate scope (up to 1.8 GHz) to record the electrical signal, capturing the fast reaction dynamics of the MBH reaction. In addition, using 2D graphene as the electrode allows the anchoring of multiple catalysts, paving the way for macroscopic synthesis at single-molecule junctions.

## RESULTS AND DISCUSSION

### Device preparation and characterization

In detail, we etched graphene on a Si/SiO_2_ chip using oxygen plasma according to the dash-line pattern, creating nanoscale gaps with carboxyl terminals [20]. This enables the integration of a carbazole-centered molecular bridge with amine terminals (Fig. 1a). The recovery of the current-voltage (*I*–*V*) response (Fig. 1b, violet curve) between source and drain electrodes indicates the bridging of the gap (open circuit, Fig. 1b, gray curve) by the molecule via covalent bonding. Under the optimized conditions, the connection yield reached ∼22% with ∼38 of 169 devices on the same chip exhibiting *I*–*V* responses (Fig. S1). Furthermore, characterization of the single-molecule electroluminescence by stochastic optical reconstruction microscopy (STORM) confirmed that the *I*–*V* responses originated from only one molecule connection [24] (Fig. 1b, inset). We then prepared the bis(dimethylaminomethyl)-substituted carbazole catalyst center (Cat) by one-step *in-situ* synthesis [25], that is, the loading of the tertiary amines for MBH catalysis (Fig. 1b, red curve). Further characterization by inelastic electron tunneling spectroscopy (IETS) verified the covalent-bond interface (amide bond) and the successful preparation of the catalytic center (Fig. 1c).

### Capturing the Michael addition intermediates

**Figure 2.**
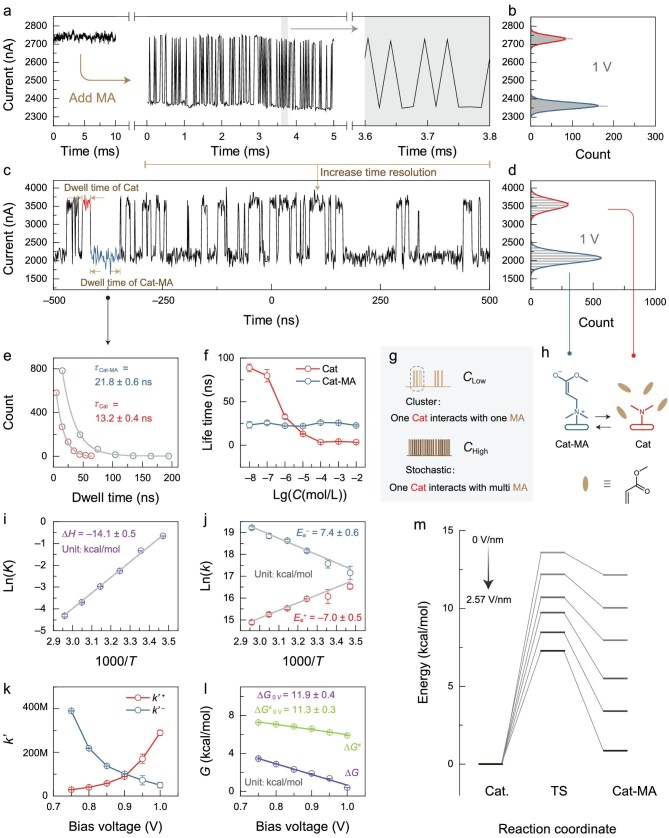
Characterization of the Michael addition reaction. (a) Left panel: the monitored *I*–*t* curve of the bare catalyst device at 298 K, 1 V bias and 54.93 kHz sampling rate, without obvious current fluctuations. Middle panel: switching between two discrete current values after adding MA (10^−3^ M). Right panel: enlarged view of the *I*–*t* curve. (b) Statistical histogram of the *I*–*t* curve in the middle panel of (a). (c) *I*–*t* curve obtained by sampling at 900 MHz in scope. (d) Statistical histogram of the *I*–*t* curve in (c). Due to the weak current signal, the histogram shows obvious quantization, which depends on the number of bits of the scope instrument. (e) Dwell times of the two conductance states extracted from (c) fitted using a single exponential decay function to obtain the corresponding lifetimes. (f) Lifetimes of Cat and Cat-MA at different concentrations of MA. (g) Schematic diagram of the current signals at different concentrations. (h) Schematic diagram of the Michael addition reaction on a single-catalyst device and the assignments of the two conductivity states. (i) Van't Hoff plot of ln*K* versus 1000/*T*, and ∆*H* obtained by linear fitting. (j) Arrhenius plots of ln*k* versus 1000/*T*, and *E* obtained by linear fitting. (k) Dependence of the forward and reverse *k*’ values on the bias voltage. (I) Dependence of the Gibbs energy on the bias voltage. The intercepts obtained from linear fitting show the results extrapolated to 0 V. (m) Computational simulations of the Michael addition reaction potential energy surfaces under different electric fields. From gray to black: 0, 0.514, 1.028, 1.542, 2.056 and 2.57 V/nm.

To study each elementary step of the MBH reaction, we firstly characterized the Michael addition reaction by adding the substrate methyl acrylate (MA). The adduct (Cat-MA, Fig. 1a) has never been captured and in its place is protonated species ((Cat-MA)H^+^) owing to the thermodynamic preference in the energy profile. In essence, as an inevitable intermediate, Cat-MA could be observed by real-time monitoring of the electrical signal. In addition, by increasing the concentration of MA, the high collision probability enables multiple reaction events of single-molecule Cat, which could be monitored at ns-scale time windows. The monitored current-time (*I*–*t*) curve with 3-μs interval sampling (439.5 kHz) at 1 V (to obtain a high current by resonant tunneling to meet the requirement of further ns-scale measurements) is shown in Fig. 2a. Specifically, after adding a super-dry dimethyl sulfoxide (DMSO) solution of MA (10^−3^ M) to the reaction cell on a chip, the observed fast binary switching (refer to the histogram in Fig. 2b) indicates multiple reaction events between Cat and Cat-MA, which was supported by the transmission spectra (Figs S2 and S3). The protonated (Cat-MA)H^+^ species is infrequently observed in a super-dry solvent and has a relatively long timescale. It was not observed under the current conditions, but it was detected in a protonic solvent (*vide infra*) and characterized by IETS (Fig. S4). However, the recorded intermediate with nearly one datum point did not elucidate the reaction dynamics. To address this issue, sampling by a scope with a ∼1 ns interval was performed (Fig. 2c), showing the switching between the two stable current levels. With similar obtained thermodynamics (histograms in Fig. 2b and d), more details of reaction dynamics were obtained. For example, by single-exponential fitting of the frequency distributions of their dwell times, the lifetimes (*τ*) of Cat and Cat-MA were determined to be 13.2 ± 0.4 ns and 21.8 ± 0.6 ns, respectively (Fig. 2e). Using *k* = 1/*τ*, the appeared rate constants *k* (i.e. conversion probabilities) were then calculated to be *k*^+^ = ∼75 M s^−1^ and *k*^−^ = ∼46 M s^−1^, respectively.

The extrapolation of single-molecule dynamics to the macroscopic level is another huge challenge owing to the uncertainty of the number of molecules involved in the intermolecular interaction and the emergent complexity with increasing scale, which has not been previously addressed. Here, the numbers of MA molecules surrounding Cat were estimated according to concentration-dependent measurements (Fig. 2f and Fig. S7). The *k* value (bimolecular process) was then corrected to compare it with the macroscopic value (*k*’). In detail, the unimolecular process of one molecule (i.e. Cat-MA → Cat + MA) can be regarded as a zero-order reaction, where *k* = *k*’. However, the *k* value of bimolecular Cat + MA → Cat-MA is influenced by the effective concentration surrounding the single-catalyst site, which requires an additional correction. Cluster-like binary switching at low concentrations was observed and showed repeated interaction between one MA molecule (regarding the concentration as ‘1’) and one Cat molecule (the details will be discussed later), where the interval between the clusters is diffusion-controlled (Fig. 2g and h). With increasing MA concentration, the descent until a constant lifetime of Cat indicates the transformation from diffusion control to dynamic control, implying a saturated MA concentration surrounding (Fig. 2g and h). According to the constant thermodynamic equilibrium of this reaction at a determined temperature, the effective number of MA under the ‘saturated’ condition was calculated to be ∼22. Therefore, the effective reaction rate constants are *k*’^+^ = ∼3.4 M s^−1^ N^−1^ and *k*’^−^ = ∼46 M s^−1^, respectively.

Based on the effective reaction rate constant, the Gibbs free energy (Δ*G*) was calculated to be ∼1.54 kcal/mol using −*RT*ln(*k*’^+^/*k*’^−^) and the energy barrier (Δ*G*^≠^) was calculated to be ∼8.51 kcal/mol using the Eyring equation. Furthermore, Δ*H* (Fig. 2i) and *E*_a_ (Fig. 2j) can be derived by temperature-dependent measurements (Fig. S8) according to Van't Hoff and Arrhenius equations, respectively. The negative dependence of *k*’^+^ on the temperature shows the highly exothermic transition state (TS), which supports the observed repeated interaction between MA and Cat to some extent, that is, the pre-reaction interaction is energetically favorable. Note that the above thermodynamic and kinetic parameters were obtained at 1 V. To extrapolate these values to a routine macroscopic condition, bias-voltage-dependent measurements were performed (Fig. S9). The results showed an exponential increase of *k*’ with the bias voltage, indicating the acceleration of the Michael addition reaction by an external electric field (EEF) (Fig. 2k). The calculated Δ*G* and Δ*G*^≠^ values showed linear relationships with the bias voltage (Fig. 2l), reminiscent of the Hammett effect. We then extrapolated the values to zero bias according to the fitted slopes. We obtained Δ*G*_0 V_ = 11.9 ± 0.4 kcal/mol and Δ*G*^≠^_0 V_ = 11.3 ± 0.3 kcal/mol, which agree with the estimated values from experiments and computational simulations [8] (Fig. 2m). The high Δ*G*_0 V_ value means that this Michael addition reaction is difficult to characterize, and the corresponding Cat-MA has not been captured at the macroscopic scale. The error of Δ*G*^≠^_0 V_ originates from the correction for concentrations around a single molecule and the assumption that the TS energy changes linearly with respect to the electric field. Here, by real-time monitoring with ns-scale time resolution, the single-molecule intermediate was detected. By extrapolation of the single-molecule dynamics to the macroscopic conditions, the thermodynamic and kinetic properties were successfully obtained.

### Correlation between Michael addition and aldol reaction

**Figure 3.**
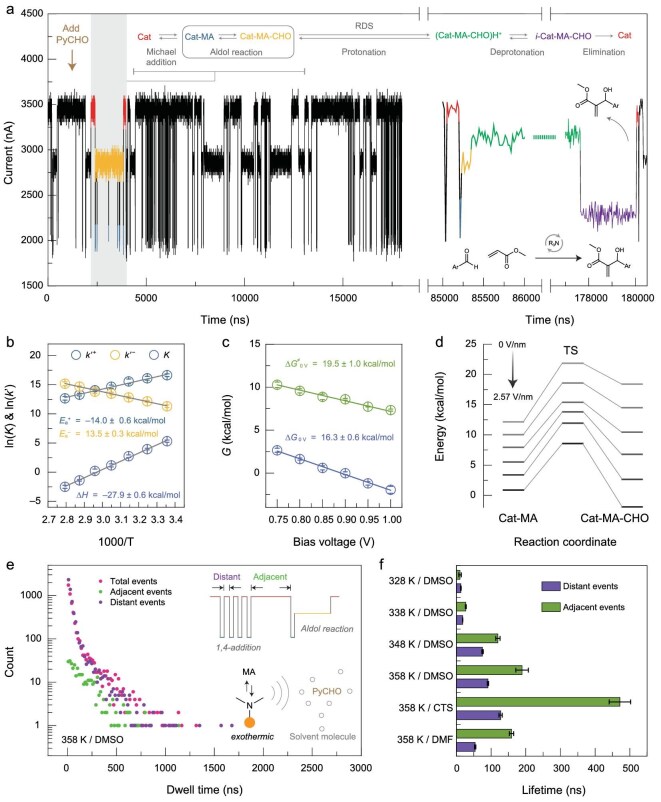
Characterization of the correlation between Michael addition and aldol reaction. (a) Left panel: monitored *I*–*t* curve after adding PyCHO (10^−3^ M) at 358 K, 1 V bias and 900 MHz sampling rate, showing Michael addition and aldol reaction. Right panel: representative catalytic cycle. The colors of the curve correspond to the species listed above the curve, showing the assignments to conductance states. (b) Van't Hoff plot of ln*K* versus 1000/T and ∆*H* obtained by linear fitting, and Arrhenius plots of ln*k* versus 1000/T and *E* obtained by linear fitting. (c) Dependence of the Gibbs energy on the bias voltage. The intercepts obtained from linear fitting show the results extrapolated to 0 V. (d) Potential energy surfaces of aldol reaction under different electric fields obtained by computational simulations. From gray to black: 0, 0.514, 1.028, 1.542, 2.056 and 2.57 V/nm. (e) Total dwell time distribution of Cat. The dwell time distributions of the events adjacent to the aldol reaction and those distant to the aldol reaction (> 6 events) are displayed, respectively. Inset: diagram of adjacent events and distant events in an ideal current signal, and the correlation between aldol reaction and 1,4-addition. (f) Lifetimes of adjacent events and distant events under different temperatures and solvent conditions.

Adding an aldehyde compound (pyridine-2-aldehyde, PyCHO) triggered the turnover of the whole catalysis cycle (Fig. 3a). Owing to the subsequent proton transfer as the rate-determining step (RDS) in DMSO, the majority of the conductance switching was among Cat, Cat-MA and Cat-MA-CHO, representing Michael addition and aldol reaction. The uncaptured Cat-MA-CHO at the macroscopic scale could be stabilized by EEF, and it was characterized by IETS (Fig. S5). The extracted thermodynamic and kinetic parameters of aldol reaction also showed a highly exothermic TS (Fig. 3b) (Δ*H^≠^* = −7.25 kcal/mol at 2.57 V/nm, Table S1). The extrapolated Δ*G*_0 V_ = 16.3 ± 0.6 kcal/mol and Δ*G*^≠^_0 V_ = 19.5 ± 1.0 kcal/mol were obtained (Fig. 3c and d) through bias-voltage-dependent measurements (Fig. S10), which explains the low yield and slow reaction rate in macroscopic synthesis and is in line with relevant theoretical simulations in the literature [8].

The real-time monitored reaction trajectories allowed us to analyze the correlation between Michael addition and aldol reaction. Continuous conversion from Cat to Cat-MA-CHO was always observed after a longer resting period of Cat. In other words, the aldol reaction exhibited a strong correlation with a longer dwell time of Cat, which was supported by the statistics of the dwell times of the distant and adjacent Cat states (Fig. 3e). Considering the highly exothermic nature of both two elementary steps (Δ*H*_Michael_ = −14.1 ± 0.5 kcal/mol (Fig. 2i) and Δ*H*_aldol_ = −27.9 ± 0.6 kcal/mol (Fig. 3b and Fig. S11)), the ‘waiting’ of Cat may be due to the boosting of the surrounding molecular diffusion [6]. By enhancing the heat dissipation and weakening the diffusion rate, decreasing the temperature strongly reduced the difference between the distant and adjacent events (Fig. 3f). In addition, solvents with different heat capacities (*c*) were used (Fig. S12). The (cyclo) tetramethylene sulfone (CTS) solvent with lower heat capacity *c*_CTS_ = 1.50 kJ/(kg·K) than DMSO (*c*_DMSO_ = 1.95 kJ/(kg·K)) showed a noticeable waiting period before continuous conversion, while *N,N*-dimethylformamide (DMF) with a higher *c*_DMF_ = 2.14 kJ/(kg·K) than DMSO exhibited the opposite result. Therefore, the two elementary steps were correlated by the energy, indicating the emergent complexity of the MBH reaction and illustrating the complex temperature dependence [26] in macroscopic synthesis to some extent.

### Clarification of the proton transfer pathways

**Figure 4.**
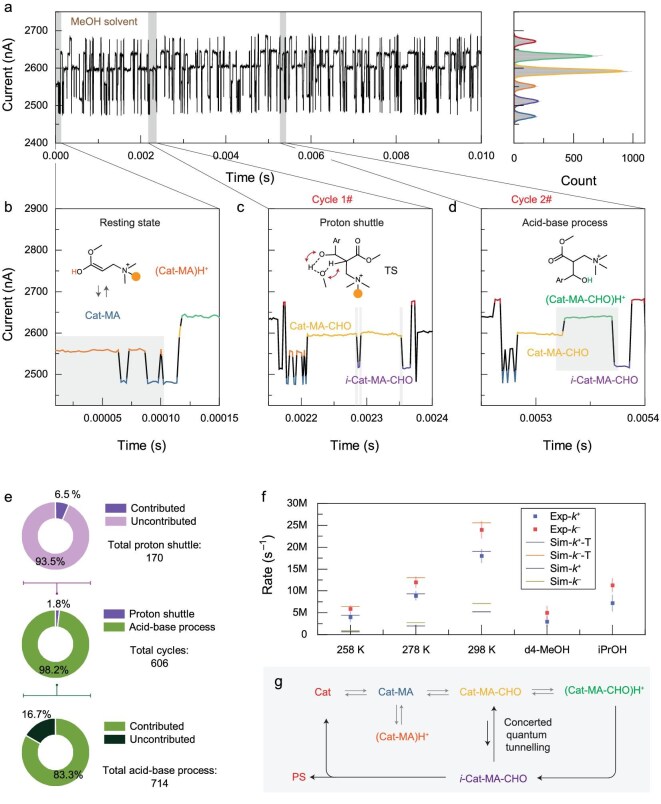
Characterization of the proton transfer paths. (a) Left panel: monitored *I*–*t* curve in a MeOH environment at 298 K, 1 V bias and 439.5 kHz sampling rate. Right panel: the corresponding statistical histogram. (b) Representative *I*–*t* curve of the resting state during catalysis. (c) Representative *I*–*t* curve of the catalytic cycle via the proton shuttle process. (d) Representative *I*–*t* curve of the catalytic cycle via the acid-base process. (e) Quantitative statistics of the contributions of the proton shuttle and acid-base processes to the complete catalysis. (f) Rate constant of the proton shuttle process obtained at 0.1 V. For temperature-dependent experiments, the uncorrected and quantum tunneling corrected computational simulations rates are provided. The experiments using d4-MeOH and *i*-PrOH as solvent were performed at 298 K. (g) Schematic diagram of the catalytic mechanism based on real-time monitoring of the chemical reaction.

Using protonic solvents significantly accelerated the subsequent proton transfer (Fig. 4a). However, there is still a debate between the proposed concerted pathway and stepwise proton transfer pathway [8,16]. Smooth catalysis cycles including the resting states of (Cat-MA)H^+^ (Fig. 4b), concerted pathway (proton shuttle) (Fig. 4c) and stepwise pathway (acid-base process) (Fig. 4d) are observed in Fig. 4a, giving a cycle period of ∼0.2 ms. The assignments to (Cat-MA)H^+^, (Cat-MA-CHO)H^+^ and *i*-Cat-MA-CHO were supported by the time sequence (Fig. 4b–d), intermediate-controlled experiments (Fig. S6), isotope experiments (Fig. S13) and transmission spectra (Fig. S2). As previously discussed, the protonation of Cat-MA was observed in MeOH solvent and showed a relatively long timescale, hindering further aldol reaction. However, external protons significantly accelerate subsequent proton transfer. The contributions of the above two pathways to the successful catalytic turnover were counted (Fig. 4e, the representative reaction trajectories are provided in Figs S14 and S15), with 170 proton shuttle events and 714 acid-base processes observed among the total 606 cycles. Eleven of the proton shuttles resulted in complete cycles, while the remaining 93.5% were reversible, returning to the original state (Cat-MA-CHO). Conversely, 83.3% of the acid-base processes contributed to complete cycles, indicating that the majority (98.2%) of the complete cycles occurred via the acid-base process. This supports the weak solvent kinetic isotope effect of this reaction and a methylation control experiment [8]. However, numerous proton shuttles were also observed in the *I*–*t* curves, despite the fact that they did not lead to a complete cycle.

The relatively high switching rates between Cat-MA-CHO and *i*-Cat-MA-CHO initiated the discussion of the concerted quantum tunneling mechanism. The obtained kinetic isotope effect (KIE) values of forward and reverse processes are in the range of 5–6, which only reaches the maximum theoretical value (Fig. 4f). This may result from the diffusion-controlled dynamics in single-molecule junctions [27]. The *i*-PrOH solvent, which has a large steric hindrance, shows slower rates relative to the methanol solvent at 298 K, indicating that quantum tunneling weakens when the barrier width is increased [28] (Fig. 4f). More importantly, relatively high rates at 258 K also suggest that concerted quantum tunneling should exist, since the calculated energy barrier should prohibit the reaction process during the detection timescale (μs) at the single-molecule level. The computational correction to the unimolecular reaction rates by quantum tunneling showed good agreement with temperature-dependent measurements (Fig. 4f and Fig. S16). Consequently, the formation of a complex between Cat-MA-CHO and the protonic solvent facilitates the concerted quantum tunneling process. However, more final states reside in Cat-MA-CHO, which is thermodynamically preferred (that is supported by the higher reverse rates than the forward rates). Therefore, the real-time observation reconciles the contradiction between the proton shuttle and acid-base processes. The protonic solvent plays crucial roles mainly in three aspects: in the resting state of the Michael addition intermediates, in the kinetic path of the proton shuttle (*vide infra*), and in the thermodynamic path of the acid-base process (which dominates the product formation and is in line with ref. [8]) (Fig. 4g). In addition, we performed electrical characterization of the MBH reaction using ethyl acrylate instead of methyl acrylate in the presence of methanol (Fig. S17). We did not observe the base-catalyzed transesterification [29] within the detection time window, probably due to the experimental settings of a single R_3_N catalyst.

### Oscillation of the MBH reaction

**Figure 5.**
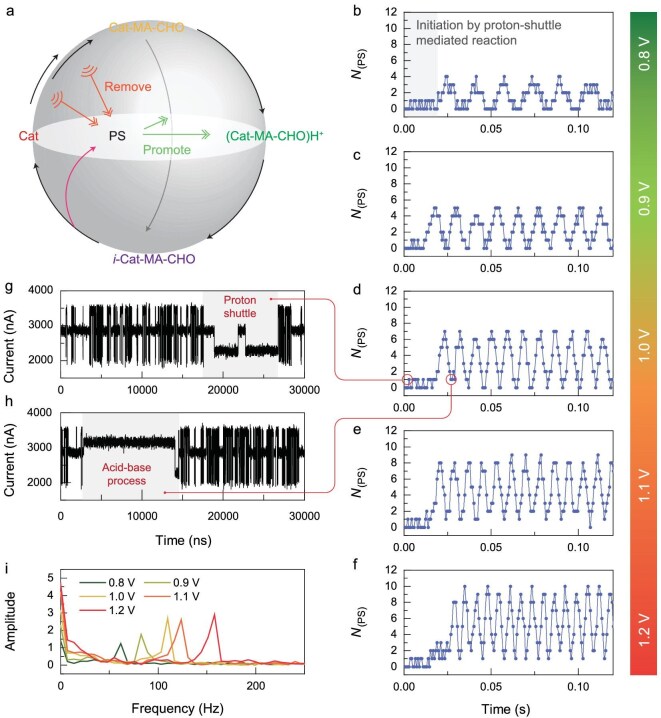
Catalytic oscillations of the MBH reaction. (a) Schematic diagram of the two feedback mechanisms present in the entire catalytic cycle. (b−f) Change of the number of generated products with time under bias voltages of 0.8–1.2 V. (g) *I*–*t* curve corresponding to a product generated during the oscillation initiation stage in (d). (h) *I*–*t* curve corresponding to a product generated during the oscillation stage in (d). (i) Frequency of the catalytic oscillation obtained through the Fourier transform.

Using an aprotic solvent (in super-dry DMSO), the MBH reaction also slowly proceeded, and it was accelerated by the well-known autocatalysis of the hydroxyl group on the product (Fig. 5a). The statistics of the formed products (with a 0.7 ms interval) revealed an abnormal reaction oscillation (Fig. 5b). Considering the highly exothermic nature of the first two elementary steps, the entire single-molecule catalytic cycle involves two feedback mechanisms. The first feedback mechanism is negative: the removal of the substrate and proton source owing to the exothermically boosted solvent diffusion from the reaction. The second feedback mechanism is positive: the autocatalysis by the formed products. These two feedback mechanisms lead to the formation of dissipative structures at the single-molecule level, where the ‘fuel’ substrate is in excess for one catalyst and the flowing electrical current provides an additional energy injection (i.e. EEF catalysis). The EEF also enhances the enthalpy changes of the first two steps (Fig. S21) and strengthens the negative feedback. Therefore, bias-voltage-dependent measurements showed an increased oscillation frequency (Fig. 5b–f), which is indicated by the Fourier transform (Fig. 5i). Consequently, an oscillator was constructed at the single-molecule level for the first time, providing the opportunity for *in-situ* drug synthesis and timed drug delivery to achieve precision medicine.

To decipher the initiation of the catalysis oscillation, the *I*–*t* curves at the initial stage are shown in Fig. 5g and h. Note that the initial several products were formed via the proton shuttle process owing to the low concentration of acidic proton donors. The direct proton transfer via a four-member ring was excluded owing to its extremally high energy barrier (Fig. S20). The reaction center outside the main electron transport channel to some extent excludes single-electron injection [30]. Therefore, once the complex forms between the reaction center and hydroxyl-containing substances (mainly *in-situ* formed products), the channel of the quantum tunnel opens, leading to the formation of a few product molecules with low probability. Monitoring in a flowing solution with substrates showed very slow product generation (Fig. S22), which supports the autocatalysis mediated by the proton shuttle process. In addition, the absence of a correlation between the PyCHO concentration and initial proton transfer rate excludes the assistance by the formation of the semi-acetal intermediate (Fig. S23) [31,32] at the single-molecule level. This may be because of the large number of proton sources and only one catalytic site. As the protons dissociate from the products produced *in-situ*, the acid-base process gradually begins and dominates the oscillation. Therefore, the proton transfer can be regarded as a kinetic path, while the acid-base process becomes a thermodynamic path, dominating the product formation.

### EEF catalysis of the MBH reaction

**Figure 6.**
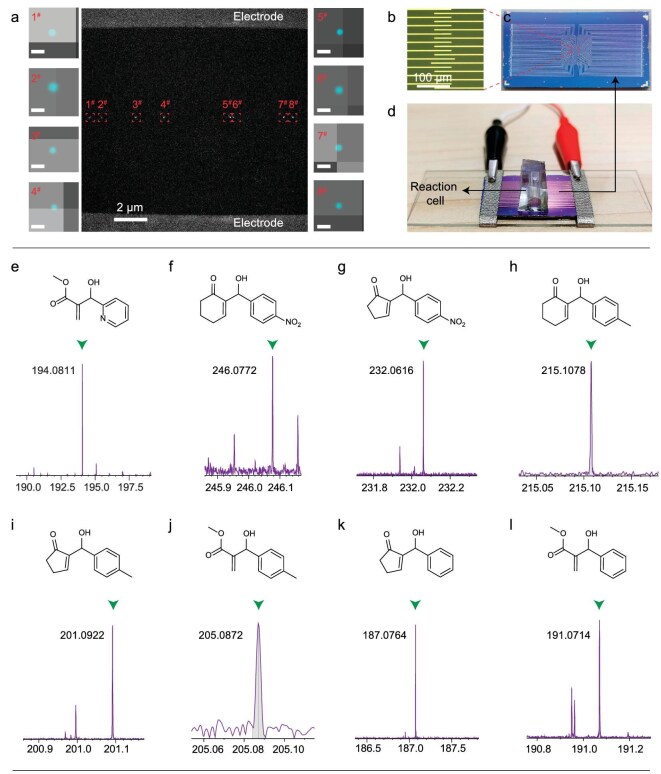
EEF catalysis of MBH reactions. (a) Eight carbazole molecular bridges anchored between a pair of metal leads and characterized by the electroluminescence. The scale bar of the eight magnification images is 30 nm. (b) Enlarged light microscope image of the single-molecule chip. (c) Photograph of a single-molecule chip. (d) Photograph of EEF catalysis for the MBH reaction on multiple single-molecule devices. (e) Mass spectrum of the solution in a reaction cell after two months catalysis by a single catalyst. (f–l) Mass spectra obtained by catalyzing different substrates through multiple devices.

The EEF flattens the reaction potential energy surface and significantly accelerates the MBH reaction (Figs S18–S20), while the 2D graphene electrodes enable the integration of multiple catalysts (Fig. 6a). As a result, the EEF catalysis on single-molecule chips can be regarded as a new synthesis paradigm. Specifically, limited by the turnover frequency (TOF) of a single catalyst (∼5000 s^−1^), despite the applied 1 V bias (the corresponding bias voltage-dependent measurements are provided in Figs S24 and S25), the single-molecule catalysis took two months to reach macroscopically detectable standards (by high-resolution mass spectrometry (HRMS), Fig. 6e). Here, by preparing a series of molecular bridges with a distribution of lengths (Fig. S26), multiple molecule integration between one pair of metal leads was approached, and it was characterized by STORM. In addition, 169 pairs of metal leads were prepared on a chip to ensure multiple catalyst integration (Fig. 6b–d). With the application of a constant bias voltage to all metal leads for 1 h, we demonstrated the effective EEF-catalysis on a single-molecule chip. Owing to the universal nature of an EEF in stabilizing polar transition states [33] and zwitterionic intermediates [34], this synthesis paradigm has a broad substrate range, including MA, 2-cyclohexen-1-one and cyclopent-2-enone as Michael addition receptors, and benzaldehyde, (electron-deficient) *p*-nitrobenzaldehyde and (electron-rich) *p*-methylbenzaldehyde as aldehyde derivatives. Within 1 h of catalysis, HRMS detectable products were obtained in all the reaction cells (Fig. 6f–i and Figs S27–S36). Therefore, we believe that with the high-density integration of single-molecule electrical devices in the future, in addition to continuing Moore's Law, on-device synthesis will gradually move to the production line.

## CONCLUSION

Determining the reaction mechanism and enhancing the rate and yield are general challenges for all chemical reactions, including the MBH reaction as described here. We have elucidated the complex mechanism of the MBH reaction by real-time monitoring of single-molecule trajectories with nanosecond-scale resolution, capturing all hidden intermediates. By extrapolating the single-molecule dynamics to macroscopic conditions, the thermodynamic and kinetic parameters were obtained. Furthermore, the observed trajectories clarified the contributions of the two proposed proton transfer pathways to the catalytic cycles, including the proton shuttle process as the kinetic path and the acid-base process as the thermodynamic path. In addition, the emerging complexity among the multiple elementary steps was revealed, including the oscillation of the catalytic cycle at the single-molecule level.

The EEF precisely regulate the MBH reaction, including lowering the energy barriers of the Michael addition and aldol reaction, which are in the ascending stage of the steep potential energy surface, and the regulation of the oscillation frequency. Based on the reaction mechanism, the EEF-catalysis was demonstrated on a single-molecule chip and achieved a TOF of ∼5000 s^−1^ at 1 V bias voltage, which addresses the challenges of the slow reaction rates and low yields of the MBH reaction.

More of the complexity of chemical reactions remains to be unveiled. For example, the highly exothermic nature of the two steps in the MBH reaction may cause interference among the catalytic cycles. The formation of a semi-acetal intermediate might play a more important role in the proton transfer in aprotic solvents. This work focuses on the turnover of one individual catalyst and provides an understanding, with insights, at the single-molecule level. The extrapolation from a single-molecule to an ensemble is still a challenge. The scalability of single-molecule electrical devices is crucial for achieving a complete understanding of the reaction mechanism and high-throughput preparation via EEF catalysis.

## Supplementary Material

nwaf172_Supplemental_File
