# Supplementary material for: Nanosecond-scale single-molecule reaction dynamics for scalable synthesis on a chip
Source: Natl Sci Rev. 2025 Apr 26;12(9):nwaf172. doi: 10.1093/nsr/nwaf172 (PMC12342527; doi:10.1093/nsr/nwaf172)
Supplement: nwaf172_Supplemental_File [file nwaf172_supplemental_file.pdf]

Supplementary data for

Nanosecond-scale single-molecule reaction dynamics for scalable synthesis on a chip

Chen Yang[1,†], Shuyao Zhou[1,†], Yilin Guo[1,†], Zexi Hou[2], Junhao Li[1], Zhirong Liu[1], Zitong Liu[3*], Deqing Zhang[4*], Yanwei Li[2*], Kendall N. Houk[5*] & Xuefeng Guo[1,6*]

[1]Beijing National Laboratory for Molecular Sciences, National Biomedical Imaging Center, College of Chemistry and Molecular Engineering, Peking University, 292 Chengfu Road, Haidian District, Beijing 100871, P. R. China.

[2]Environment Research Institute, Shandong University, Qingdao 266237, P. R. China.

[3]State Key Laboratory of Applied Organic Chemistry, College of Chemistry and Chemical Engineering, Lanzhou University, Lanzhou, Gansu 730000, P. R. China

[4]Beijing National Laboratory for Molecular Sciences, CAS Key Laboratory of Organic Solids, Institute of Chemistry, Chinese Academy of Sciences, Beijing 100190, P. R. China

[5]Department of Chemistry and Biochemistry, University of California, Los Angeles, Los Angeles, CA 90095-1569, USA.

[6]Center of Single-Molecule Sciences, Frontiers Science Center for New Organic Matter, College of Electronic Information and Optical Engineering, Nankai University, 38 Tongyan Road, Jinnan District, Tianjin 300350, P. R. China.

[†]These authors contributed equally to this work.

*Correspondence to: guoxf@pku.edu.cn (X.G.); houk@chem.ucla.edu (K.N.H.); lyw@sdu.edu.cn (Y.L.); dqzhang@iccas.ac.cn (D.Z.); liuzt@lzu.edu.cn (Z.L.)

Table of content

1. **Materials and methods**
2. **Characterization of the single-molecule device**
3. **The assignments of the conductance states**
4. **Characterization of the first step of the MBH reaction**
5. **Characterization of the first two steps of the MBH reaction**
6. **Characterization of the proton transfer of the MBH reaction**
7. **Reaction potential energy surface involving different proton transfer mechanisms**
8. **Characterization of the single-molecule catalytic oscillations**
9. **Bias dependence of the whole MBH reaction**
10. **Characterization of the macroscale synthesis**

1. Materials and methods

Molecular synthesis and characterization

The reagents and starting materials were commercially available and used without any further purification, if not specified elsewhere. Compounds **1** and **2** were synthesized according to the previous report [1,2]. ^{1}H NMR spectra were recorded on Bruker AVANCE III 400, 500 and 600 MHz spectrometers. Mass spectra were determined with a Bruker Solarix-XR high-resolution mass spectrometer.

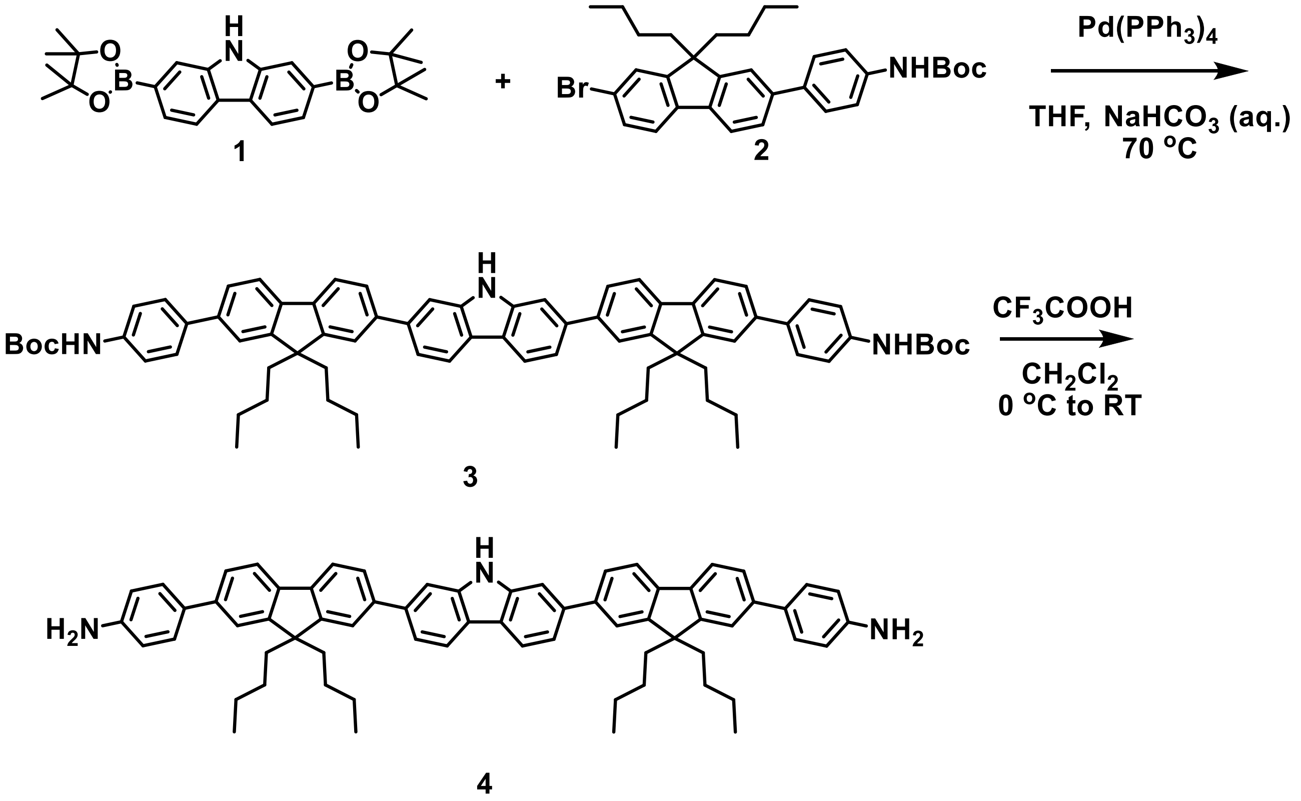

<u>Synthesis of **3**</u>

To a Schlenk flask equipped with a stir bar was added compound **1** (75.4 mg, 0.18 mmol) and compound **2** (200 mg, 0.36 mmol). The vessel was sealed and evacuated/backfilled with nitrogen for three times, followed by the addition of THF (20 mL) and $NaHCO_3$ saturated aqueous solution (5 mL) via a syringe. $Pd(PPh_3)_4$ was added in one portion (41.6 mg, 50 μmol). The vessel was charged with nitrogen through a freeze-pump-thaw cycle for three times. Then, the mixture was heated at reflux for 12 h. After cooling down, solvents were removed by rotary evaporation and the residue was purified by column chromatography with CH_2Cl_2 and petroleum ether (60–90 °C) (1:5 to 1:1, v/v) as the eluent. Compound **3** was

obtained as an off-white solid (~92 mg) in ~46% yield. ^1H NMR ($CDCl_3$, 400 MHz): δ 8.23 (s, 1H), 8.18 (d, J = 8.1 Hz, 2H), 7.81 (d, J = 7.8 Hz, 2H), 7.78 (d, J = 7.8 Hz, 2H), 7.75 (d, J = 1.5 Hz, 2H), 7.72-7.71 (m, 2H), 7.69 (m, 2H), 7.63 (m, 2H), 7.62 – 7.60 (m, 4H), 7.58-7.57 (m, 2H), 7.55 (m, 2H), 7.47 (d, J = 8.1 Hz, 4H), 6.54 (s, 2H), 2.11 – 2.05 (m, 8H), 1.55 (s, 18H), 1.26 (m, 8H), 1.13 (q, J = 7.4 Hz, 8H), 0.71 (t, J = 7.3 Hz, 12H). HRMS (MALDI-TOF) m/z: calcd for $C_{76}H_{83}N_3O_4$ $[M+Na]^+$, 1124.6276; found, 1124.6266.

Synthesis of compound **4**

To a Schlenk flask equipped with a stir bar was added compound **3** (80 mg, 0.07 mmol), and CH_2Cl_2 (10 mL). After cooling down to 0 ^oC, trifluoroacetic acid (3 mL) was slowly added. The vessel was charged with nitrogen for three times. Then, the mixture was stirred for 5 h under nitrogen atmosphere. K_2CO_3 (2 M) was slowly added to the system until pH reaching 8 under 0 ^oC. The system was poured into water. The organic phase was extracted by ethyl acetate (EA, 3 × 20 mL); the organic layer then was washed with saturated brine, dried over sodium sulfate, and evaporated to dryness. The residue was purified by column chromatography with CH_2Cl_2 and EA (100:1, v/v) as the eluent. Compound **4** was obtained as a gray foam solid (~60 mg) in ~90% yield. ^1H NMR ($CDCl_3$, 400 MHz): δ 8.19 (s, 1H), 8.18 (d, J = 2.4 Hz, 2H), 7.81 (m, 4H), 7.79 (d, J = 2.0 Hz, 2H), 7.77 (s, 2H), 7.74 – 7.73 (m, 2H), 7.63 (s, 2H), 7.61 – 7.60 (m, 2H), 7.57 (m, 4H), 7.55 (s, 2H), 7.52 (m, 2H), 7.50 (m, 2H), 6.81 (s, 2H), 6.78 (s, 2H), 2.10 (m, 8H), 1.27 (m, 8H), 1.14 (m, 8H), 0.71 (t, J = 7.2 Hz, 12H).. HRMS (MALDI-TOF) m/z: calcd for $C_{66}H_{67}N_3$ $[M+H]^+$, 902.5413; found, 902.5408.

Preparation of the single-molecule catalyst devices

A 25 μm-thick copper sheet was firstly pretreated with acetic acid to etch the surface oxide layer, followed by sequential rinsing with deionized water and ethanol, and final drying with N_2. Subsequently, single-layer graphene was synthesized on the pretreated copper sheet by a chemical vapor deposition process. The copper-substrate graphene was then coated with a layer of polymethyl methacrylate (PMMA) 950 via spin-coating to form the PMMA-graphene-copper layers. The copper substrate was then removed using an $FeCl_3$ solution, resulting in the transfer of graphene from the copper substrate to PMMA. The PMMA-supported graphene was then transferred to a pretreated silicon wafer (3.8 cm × 2.5 cm) with a 300 nm SiO_2 layer through successive rinsing with HCl solution and deionized water. After removing the PMMA layer with acetone, photolithography and thermal evaporation were performed to deposit gold marks on the graphene-silicon wafer. Subsequently, a graphene ribbon with a width of 40 μm was prepared by photolithography and oxygen plasma etching. Successive evaporation of 8 nm Cr and 60 nm Au was performed on the Si wafer to serve as the metal leads. A 40 nm SiO_2 layer was then deposited by electron beam evaporation to prevent current leakage in the liquid phase. Using the dash-line lithographic method, the graphene FET arrays were spin-coated with a layer of PMMA and exposed via electron beam lithography to open windows on the PMMA layer. Finally, nanogaps with carboxyl terminals between the graphene point contact electrodes were obtained through oxygen plasma etching and electrical burning.

The freshly prepared graphene device was placed in a round-bottom flask containing a 0.1 mM molecular bridge and 1 mM 1-(3-dimethylaminopropyl)-3-ethylcarbodiimide hydrochloride in 10 mL of pyridine. Following a 48-h condensation reaction period, the device was removed, thoroughly rinsed with deionized water and acetone, and dried with N_2. The device was then characterized by $I-V$ scanning. The recovery of the $I-V$ response indicated successful integration of the molecular bridge into graphene electrodes. To load the R_3N catalyst center, 1 mM bis(dimethylamino)methane, 1 mM K_2CO_3 and 1 mM succinic

anhydride in 10 mL of CH_2Cl_2 was then added into a round-bottom flask with a single-molecule carbazole device. After 1 h, the device was removed, thoroughly rinsed with CH_2Cl_2 and dried with N_2. The successful immobilization of R_3N catalyst center was verified by *I*−*V* scanning and IETS.

Electrical characterization

The single-molecule device was placed in a probe station (Lake Shore TTPX) during the experiments. The *I*–*V* curves were measured with an Agilent B1500A semiconductor parameter system. The output terminal of the UHFLI lock-in amplifier provided a constant bias for the *I*–*t* measurement. The current signal of the molecular loop was amplified by a DHPCA-100 pre-amplifier with 200MHz band width and then recorded by an oscilloscope.

Theoretical calculations

The geometry optimization and frequency vibrational computations were performed with the Gaussian 16 program [3] M06-2X/6-31G(d) level, and the single-point energies were calculated at M06-2X/6-311++g(3df,3pd) level [4]. The external electric field was applied along the molecular bridge in the experiment and for the structures of other species, the direction of the external electric field was set in the same way. The SMD implicit solvation model was adopted to simulate the dimethylsulfoxide solvent environment [5]. With the aid of software Shermo [6], the thermodynamic data required for calculation were generated. The expression for the reaction rate constant based on transition state theory is

$$k = \sigma \frac{k_B T}{h} e^{-\Delta G^{0,\neq}/(k_B T)} \quad (1)$$

where σ is the degeneracy of the reaction path, k_B is the Boltzmann constant, T is the temperature, h is the Planck constant, and $\Delta G^{0,\neq}$ is the activation free energy of the standard state. After considering the tunnelling effect, the transmission coefficient κ was introduced into Eq. (1) to give Eq. (2):

$$k = \kappa\sigma \frac{k_B T}{h} e^{-\Delta G^{0,\neq}/(k_B T)} \quad (2)$$

κ was obtained by using the Skodje-Truhlar method, i.e., the small curvature tunneling (SCT) approximation [7]. The small curvature approximation is a commonly used method for multidimensional tunneling calculations in computational chemistry [8]. The influence of the tunneling effect on the rate constant is given by $k_{CVT+SCT} = \kappa_{SCT}\, k_{CVT}$, where k_{CVT} is the rate constant excluding the tunneling effect. According to the Canonical variational transition state theory (CVT), κ_{SCT} is the transmission coefficient of SCT, while $k_{CVT+SCT}$ is the rate constant including the tunneling effect of SCT. κ_{SCT} can be obtained according to the Bell and Truhlar functions, which contain the temperature T, Boltzmann constant k_B, Planck's constant h, imaginary frequency $i\nu^{\neq}$, and barrier height V_0. Therein, $i\nu^{\neq}$ and V_0 were obtained with explicit simulations including geometry optimization and vibrational frequency calculations of the molecular bridge. Specifically:

$$\kappa(\mathrm{T}) \cong \frac{\pi\beta/\alpha}{\sin(\pi\beta/\alpha)} + \frac{\beta}{\beta-\alpha}\, e^{(\beta-\alpha)V_0} \qquad (\alpha > \beta)$$

$$\kappa(\mathrm{T}) = \alpha V_0 = \beta V_0 \qquad (\alpha = \beta)$$

$$\kappa(\mathrm{T}) \cong \frac{\beta}{\beta-\alpha}\{\exp[(\beta-\alpha)V_0] - 1\} \qquad (\alpha < \beta)$$

Therein:

$$\alpha = 2\pi/h\nu^{\neq}$$
$$\beta = 1/k_B T$$

Then, we obtained all the κ at different temperatures and the corresponding k_{CVT}, which were provided in Fig. 4f.

The transport properties of these molecular junctions were implemented using a combination of DFT and non-equilibrium Green's function method (NEGF) within the Atomistix toolkit (ATK) package [9]. The general gradient approximation was used, double ζ polarized basis set and pseudodojo pseudopotential were adopted, and the energy cut off was set as 100 Hartree. The k-point sampling was set as 24×1 for the calculation of transmission spectra.

2. Characterization of the single-molecule device

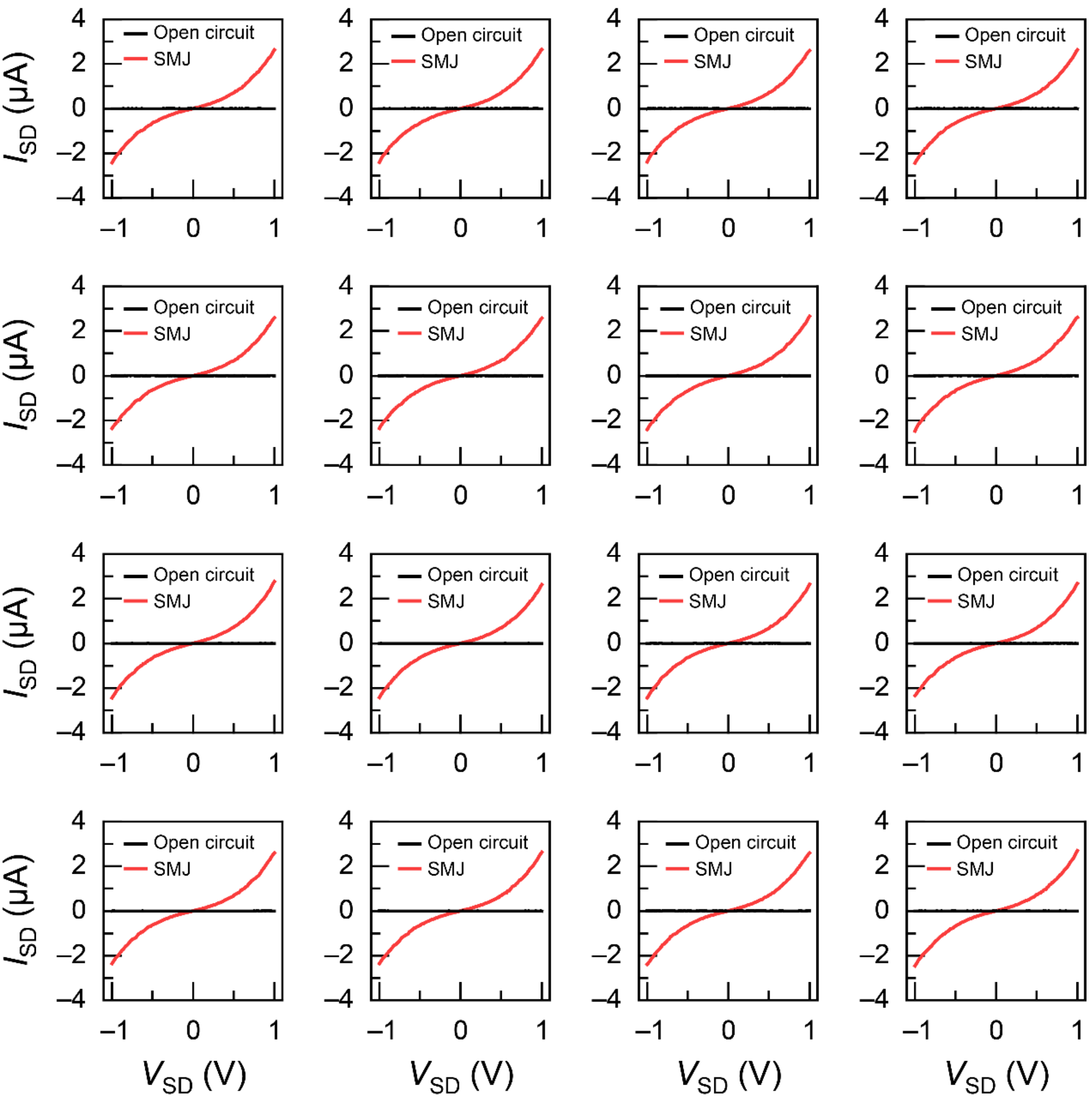

Figure S1.16 *I*−*V* curves of carbazole-based single-molecule junctions. The successful preparation of single-molecule junctions was determined by comparing the current-voltage (*I*−*V*) relation curve after oxygen plasma etching (open circuit, black line) with the curve after connecting the molecules between metal electrodes (recovered to some extent, red line).

3. The assignments of the conductance states

As discussed in the main text, the assignments of conductance states can be supported by transmission spectra, IETS, multiple control experiments, and theoretical simulations. Specifically, the bare Cat can be characterized by IETS; Cat-MA, which has the lowest conductance, has a lifetime on the ns scale and can be verified by addition of MA. This is a new conductance state with MA addition only, and its lifetime is independent on MA concentration. But its occupancy increases with increasing MA concentration. (Cat-MA)H$^+$ is a stable state, which can be characterized by IETS; Cat-MA-CHO, an intermediate of the subsequent reaction, is also stable and can be verified by IETS; i-Cat-MA-CHO is a binding state between the catalyst and the product. In-situ observation was conducted by adding products (intermediate control experiment); For (Cat-MA-CHO)H$^+$, the conductance state is assigned by the solvent effect due to the proton transfer involved. The conductance state hardly appears in aprotic solvents, and the lifetime of the conductance state becomes longer in the presence of d4-CH$_3$OH, which proves the correctness of the signal assignments.

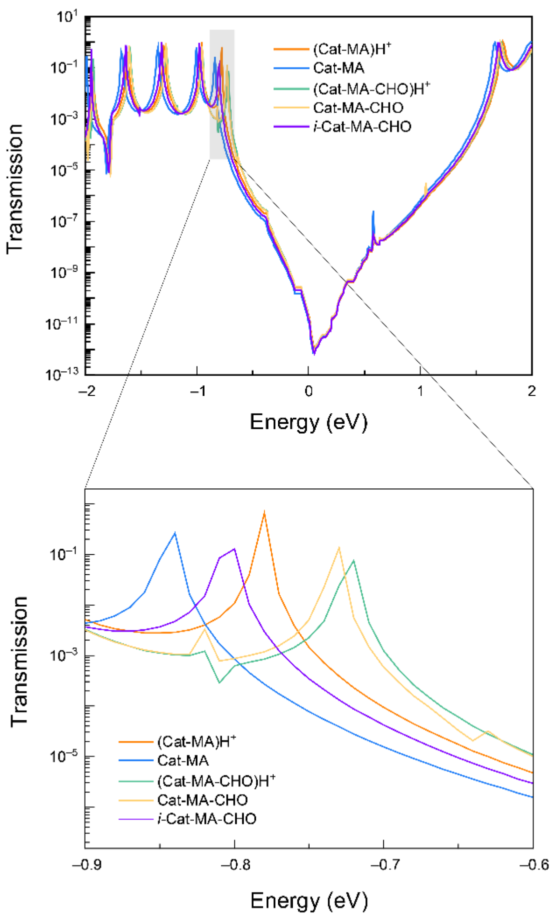

Figure S2. Transmission spectra of the observed various intermediates and enlarged views of the main transmission peaks. According to the order of distance between the transmission peak and the Fermi level, the conductance from high to low are (Cat-MA-CHO)H$^+$, Cat-MA-CHO, (Cat-MA)H$^+$, *i*-Cat-MA-CHO, and Cat-MA, respectively.

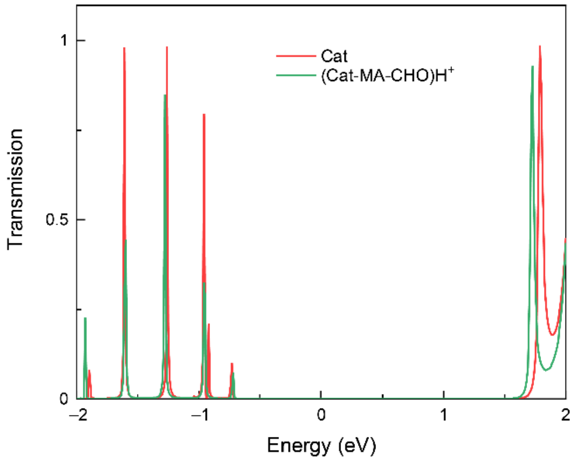

Figure S3. Comparison of the transmission spectra of the initial Cat State (without adding substrate reactant) with the highest transmittance ((Cat-MA-CHO)H$^+$) during the reaction. The Cat has a higher transmittance than all other species. For convenience it is compared alone with the second highest intermediate. The Cat shows a higher transmission peak (red line), supporting the assignment of all the six species.

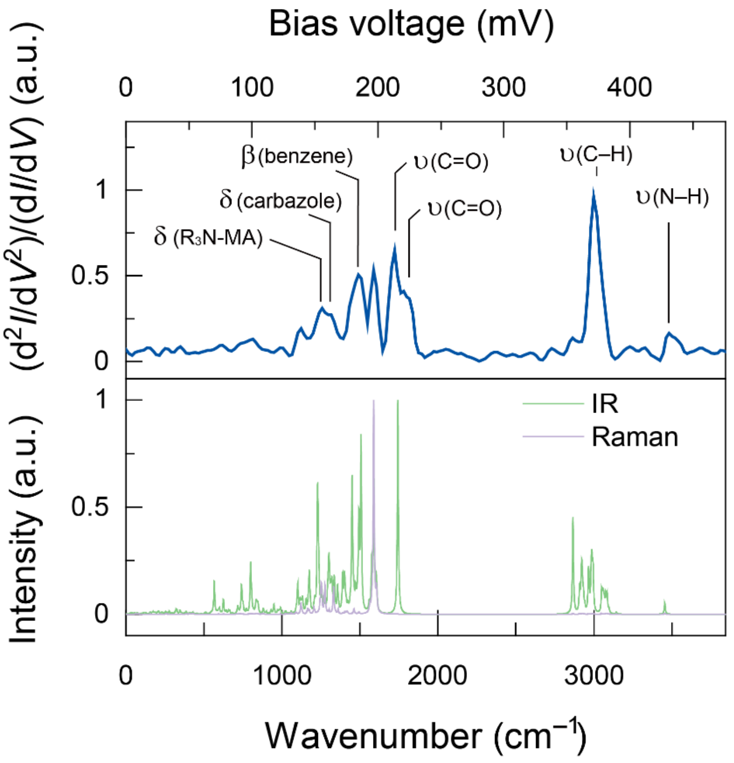

Figure S4. IETS of the (Cat-MA)H$^+$ intermediate. The IETS was characterized at 2 K with an AC modulation of 21.2 mV at a frequency of 661 Hz. Bottom: simulated infrared and Raman spectra of the corresponding molecular bridge. The peaks assigned to specific vibrational modes are marked out in the IETS ($V = h\omega/e$). We observed the peaks of δ (carbazole) (~160 mV), v (C−H) (350~380 mV), and δ (R_3N-MA) (~155 mV). In addition, the specific peaks of v(C=O) (~213 mV and ~220 mV) of MA moiety and amide bond as well as v(N−H) (~425 mV) in the amide bond can be detected, respectively.

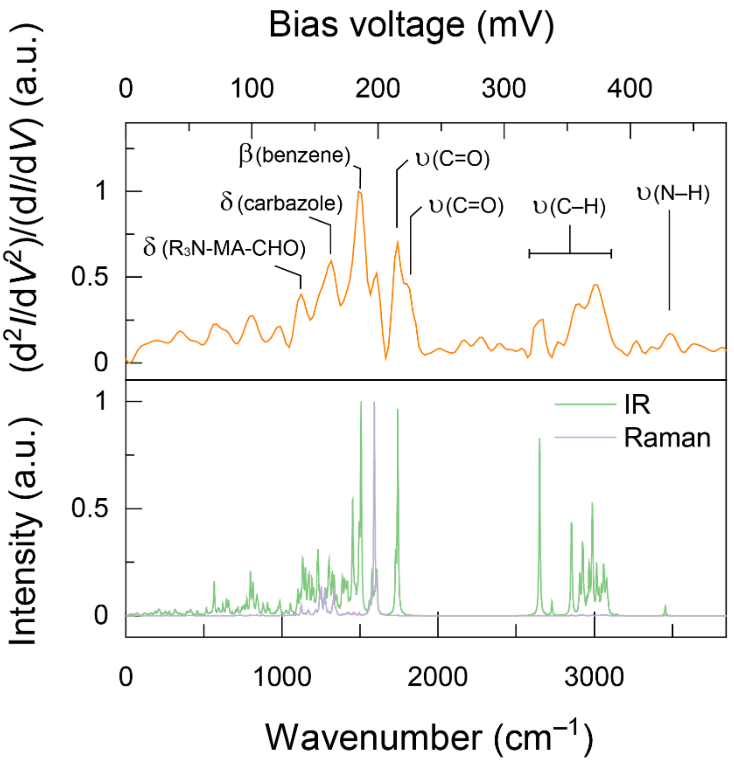

Figure S5. IETS of the Cat-MA-CHO intermediate. The IETS was characterized at 2 K with an AC modulation of 21.2 mV at a frequency of 661 Hz. Bottom: simulated infrared and Raman spectra of the corresponding molecular bridge. The peaks assigned to specific vibrational modes are marked out in the IETS ($V = h\omega/e$). We observed the peaks of δ (carbazole) (~160 mV), v (C−H) (350~380 mV) and δ (R_3N-MA-CHO) (~137 mV). In addition, the specific peaks of v(C=O) (~215 mV and ~223 mV) of MA moiety and amide bond as well as v(N−H) (~425 mV) in the amide bond can be detected, respectively.

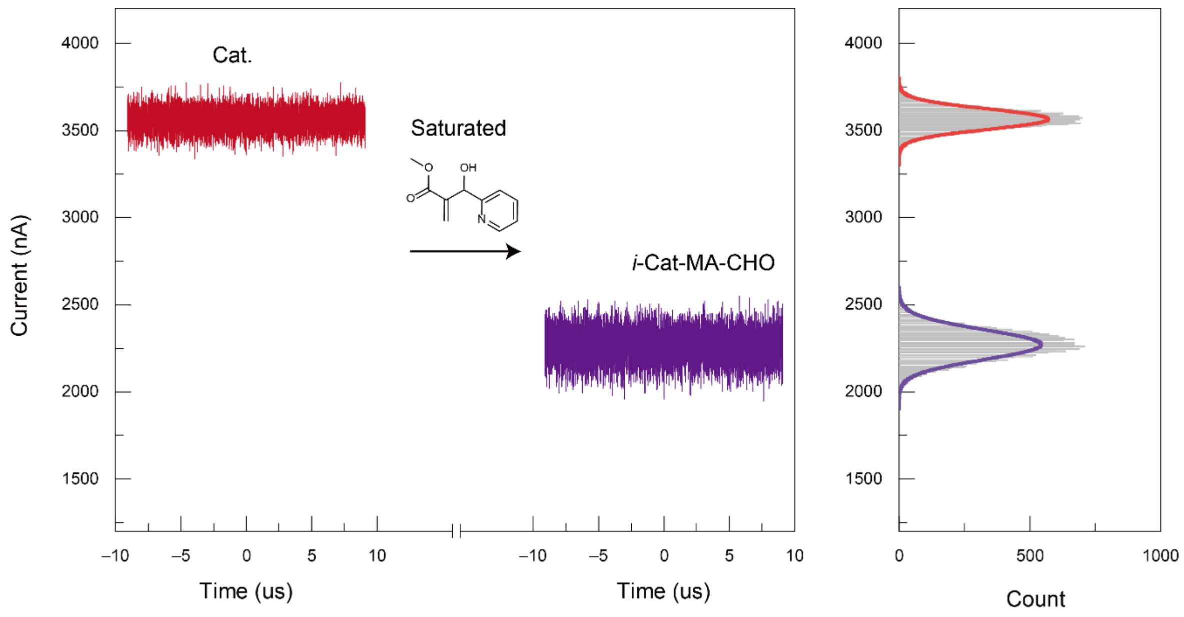

Figure S6. Intermediate-controlled experiments at 1 V and 298 K. *I*−*t* curves and corresponding statistical histograms of the single molecule after adding saturated products methyl 2-(hydroxy(pyridin-2-yl)methyl)acrylate. By comparing the statistical histograms with Fig. 3a, the intermediate *i*-Cat-MA-CHO can be assigned.

4. Characterization of the first step of the MBH reaction

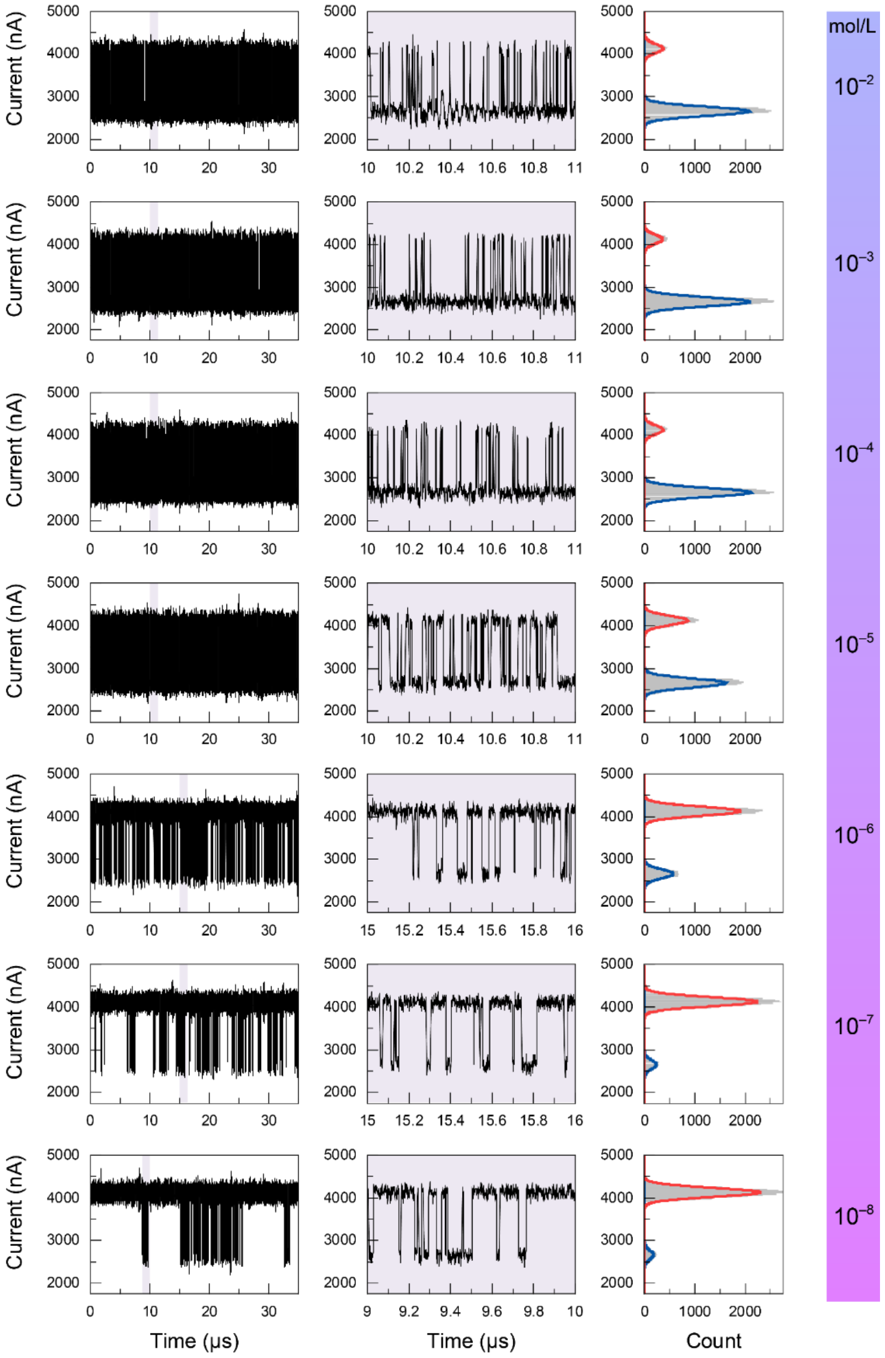

Figure S7. Concentration-dependent experiments of the first step (Michael addition reaction) at 298 K and 1 V. *I*−*t* curves, enlarged images, and corresponding statistical histograms of another single-molecule device at different concentrations of methyl acrylate (MA). Increased concentrations of MA lead to a higher reaction probability, thus the occupancy of Cat-MA state gradually increases.

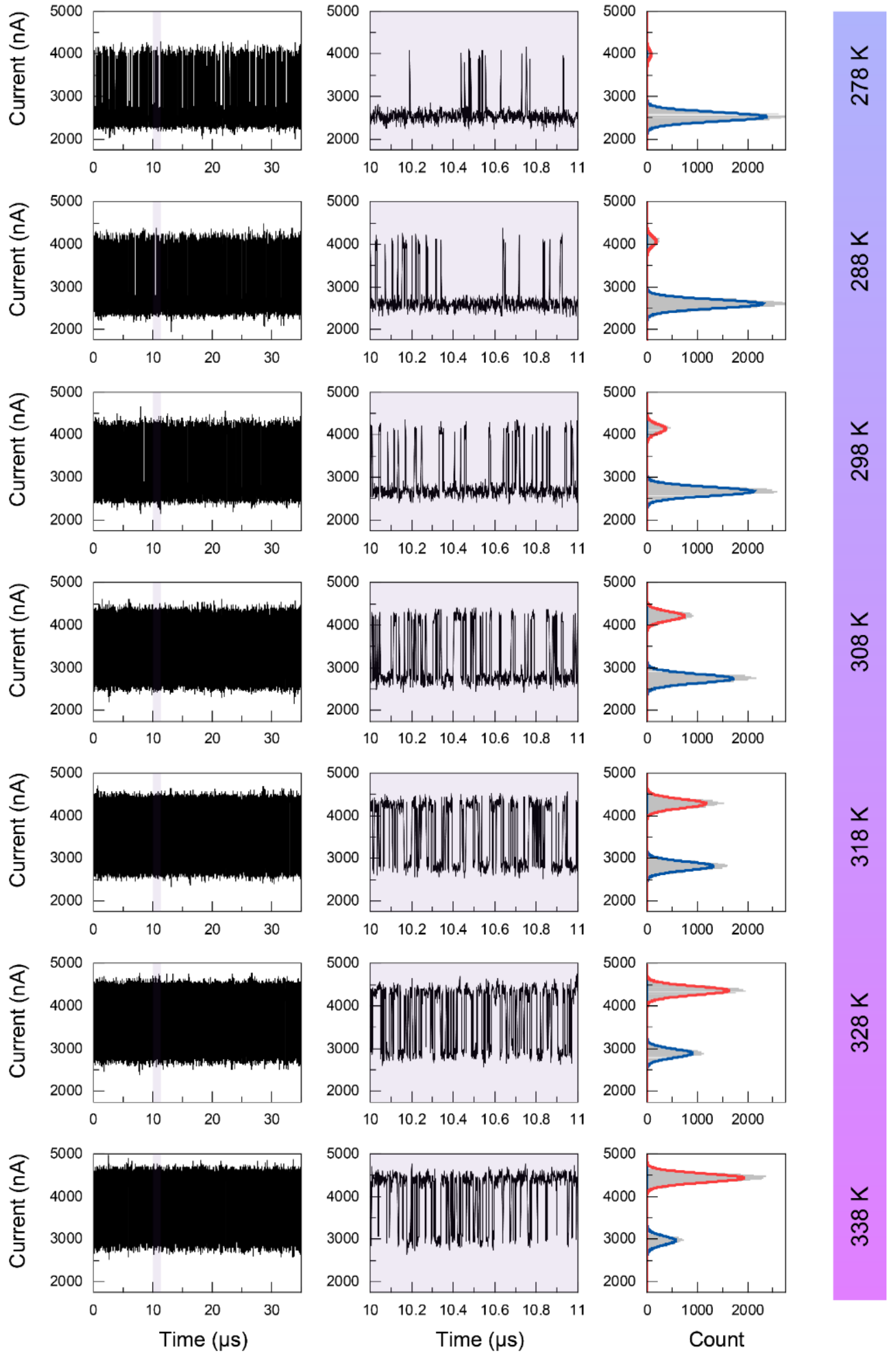

Figure S8. Temperature-dependent experiments of the first step (Michael addition reaction) at 1 V. *I*−*t* curves, enlarged images, and corresponding statistical histograms of the single-molecule device at different temperatures with adding a DMSO solution of 10^{-3} mol/L MA. With the increase of temperature, the occupancy of products (Cat-MA) decreased gradually, indicating the exothermic property of this reaction.

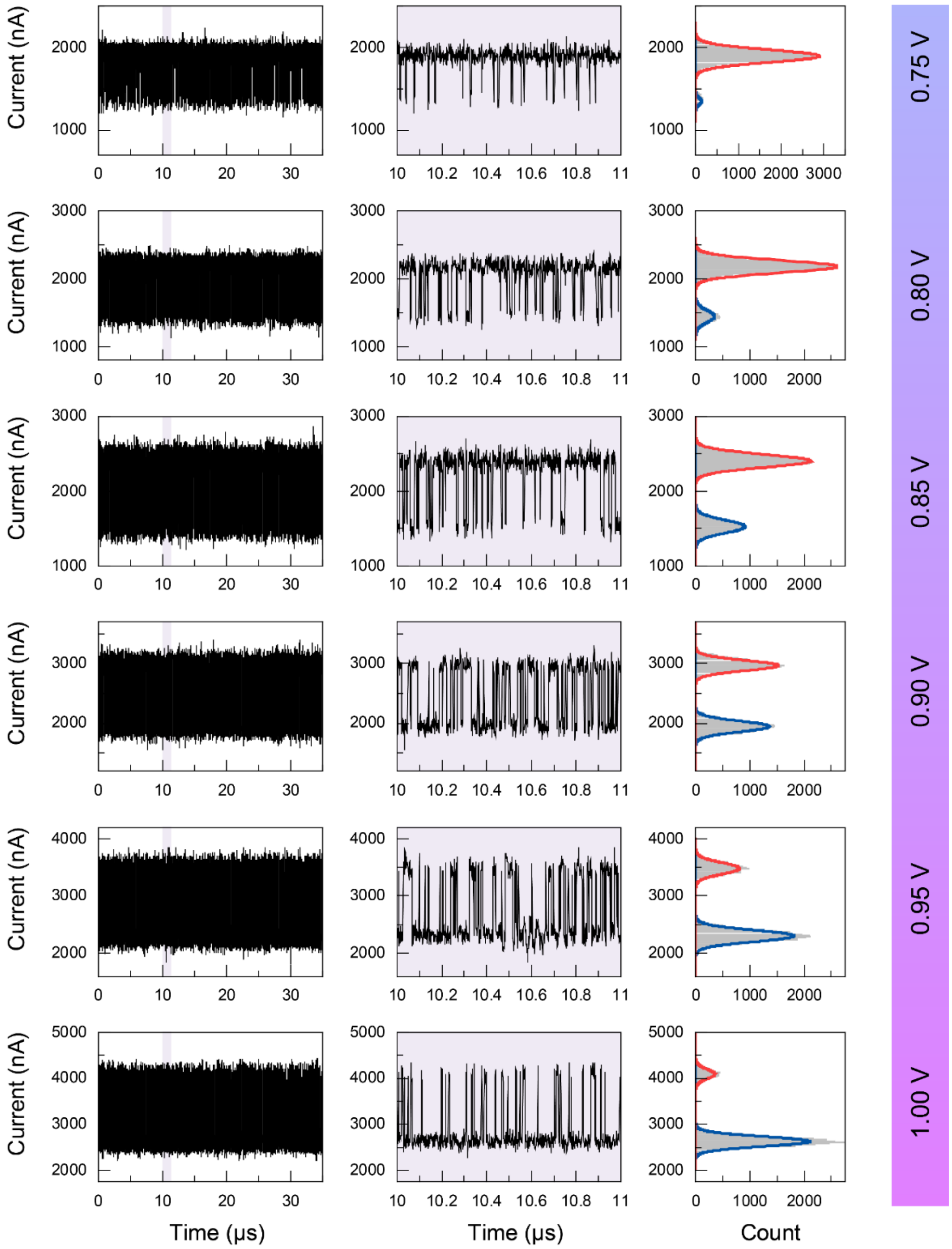

Figure S9. Bias voltage-dependent experiment of the first step (Michael addition reaction) at 298 K. *I*−*t* curves, enlarged images, and corresponding statistical histograms of the single-molecule device at different bias voltages with addition a DMSO solution of 10^{-3} mol/L MA. With the increase of bias voltage, the reaction energy barrier decreases and the reaction rate increases. At the same time, the occupancy of Cat-MA increased. This shows that the electric field effectively drives the reaction.

5. Characterization of the first two steps of the MBH reaction

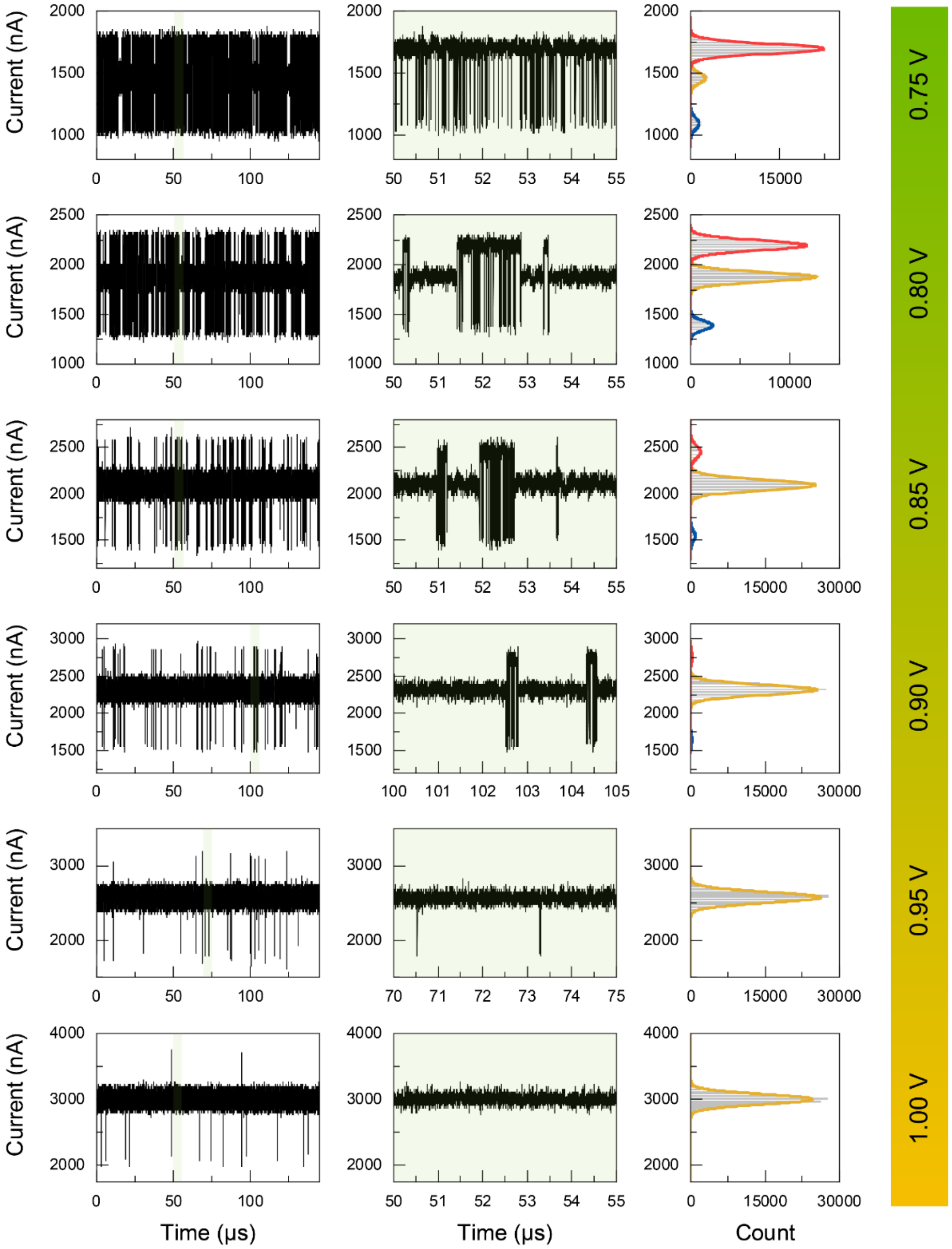

Figure S10. Bias voltage-dependent experiments of the first two steps (Michael addition and Aldol reaction) at 298 K. *I*−*t* curves, enlarged images and corresponding statistical histograms of the single-molecule device at different bias voltages with adding a DMSO solution of 10^{-3} mol/L MA and 10^{-3} mol/L PyCHO. With the increase of bias voltage, the occupancy of Cat-MA-CHO products increased, indicating that the electric field effectively drove these two steps.

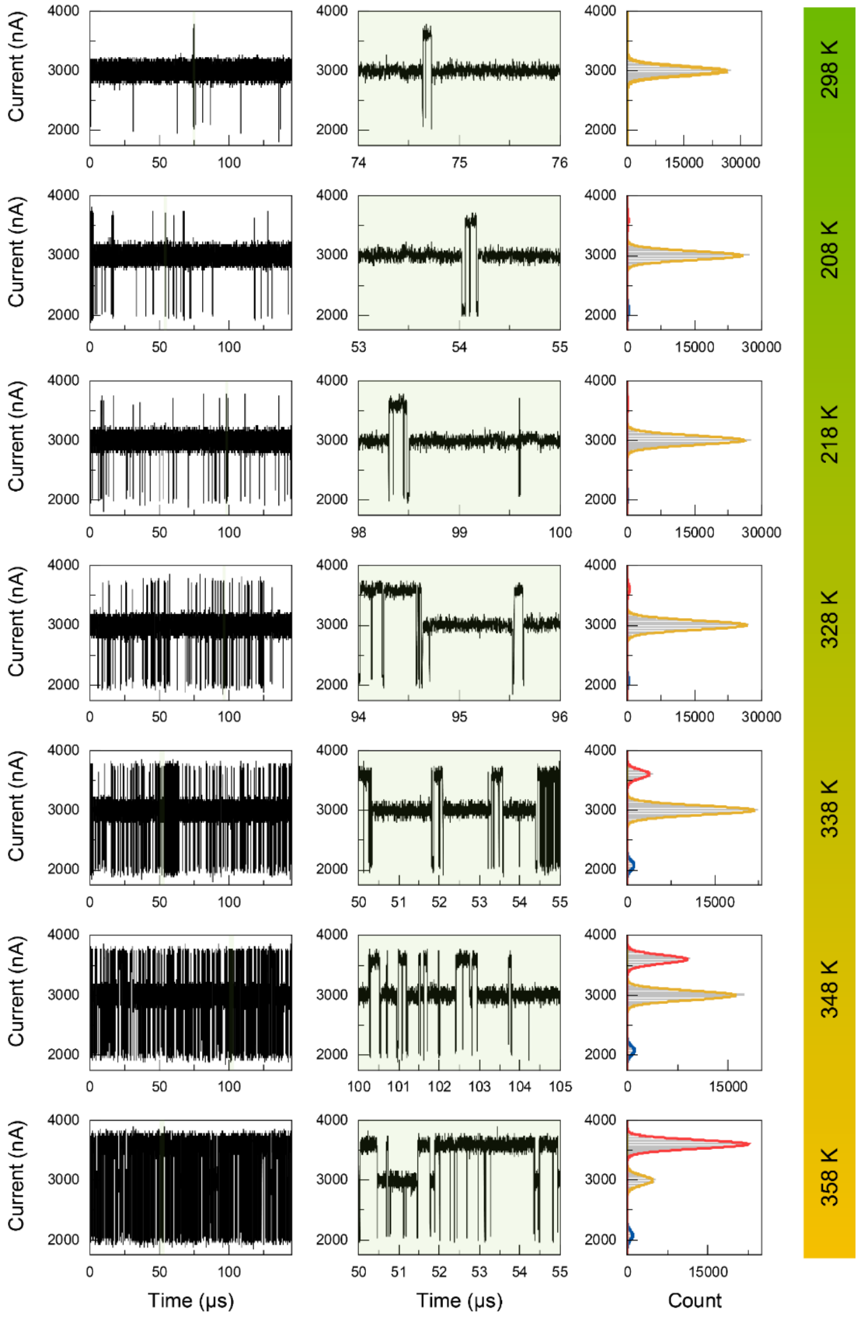

Figure S11. Temperature-dependent experiments of the first two steps (Michael addition and Aldol reaction) at 1 V. *I*−*t* curves, enlarged images and corresponding statistical histograms of the single-molecule device at different temperatures with adding a DMSO solution of 10^{-3} mol/L MA and 10^{-3} mol/L PyCHO. With the increase of temperature, the occupancy of substrate Cat increased, indicating that both steps were exothermic.

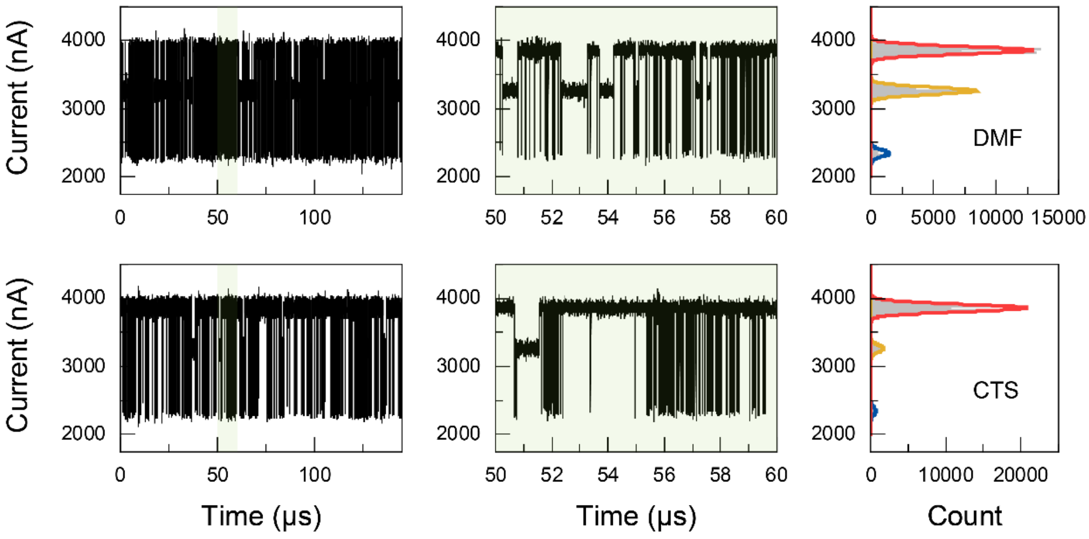

Figure S12. Solvent-dependent experiments of the first two steps (Michael addition and Aldol reaction) at 1 V and 358 K. *I*−*t* curves, enlarged images and corresponding statistical histograms of the single-molecule device at 358 K with adding a DMF or cyclo-tetramethylene sulfone (CTS) solution of 10^{-3} mol/L MA and 10^{-3} mol/L PyCHO.

6. Characterization of the proton transfer of the MBH reaction

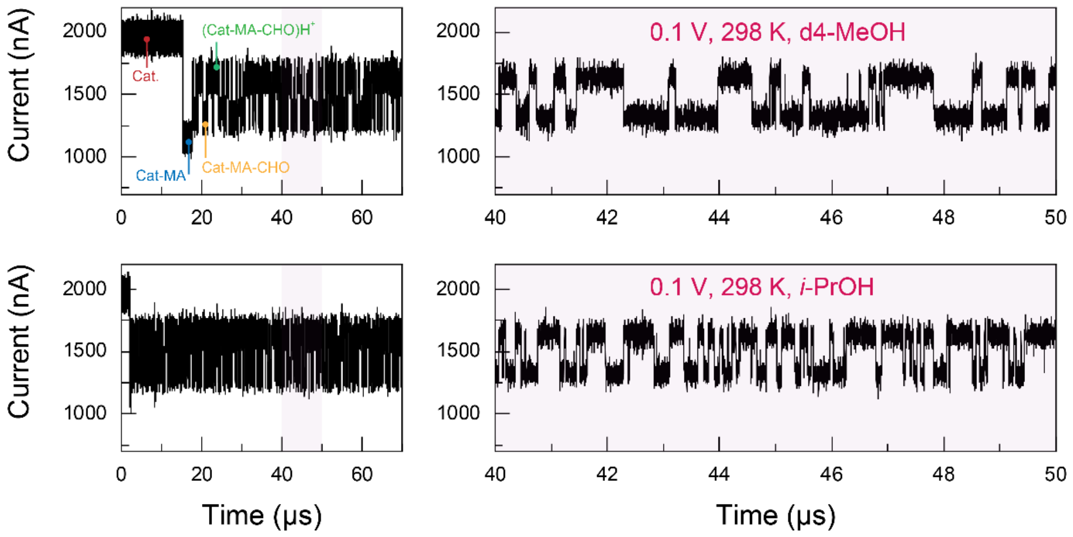

Figure S13. Solvent-dependent experiments of the proton shuttle at 0.1 V. *I*−*t* curves for proton transfer in d4-MeOH and *i*-PrOH are provided. The corresponding kinetic information in Fig. 4f is extracted here.

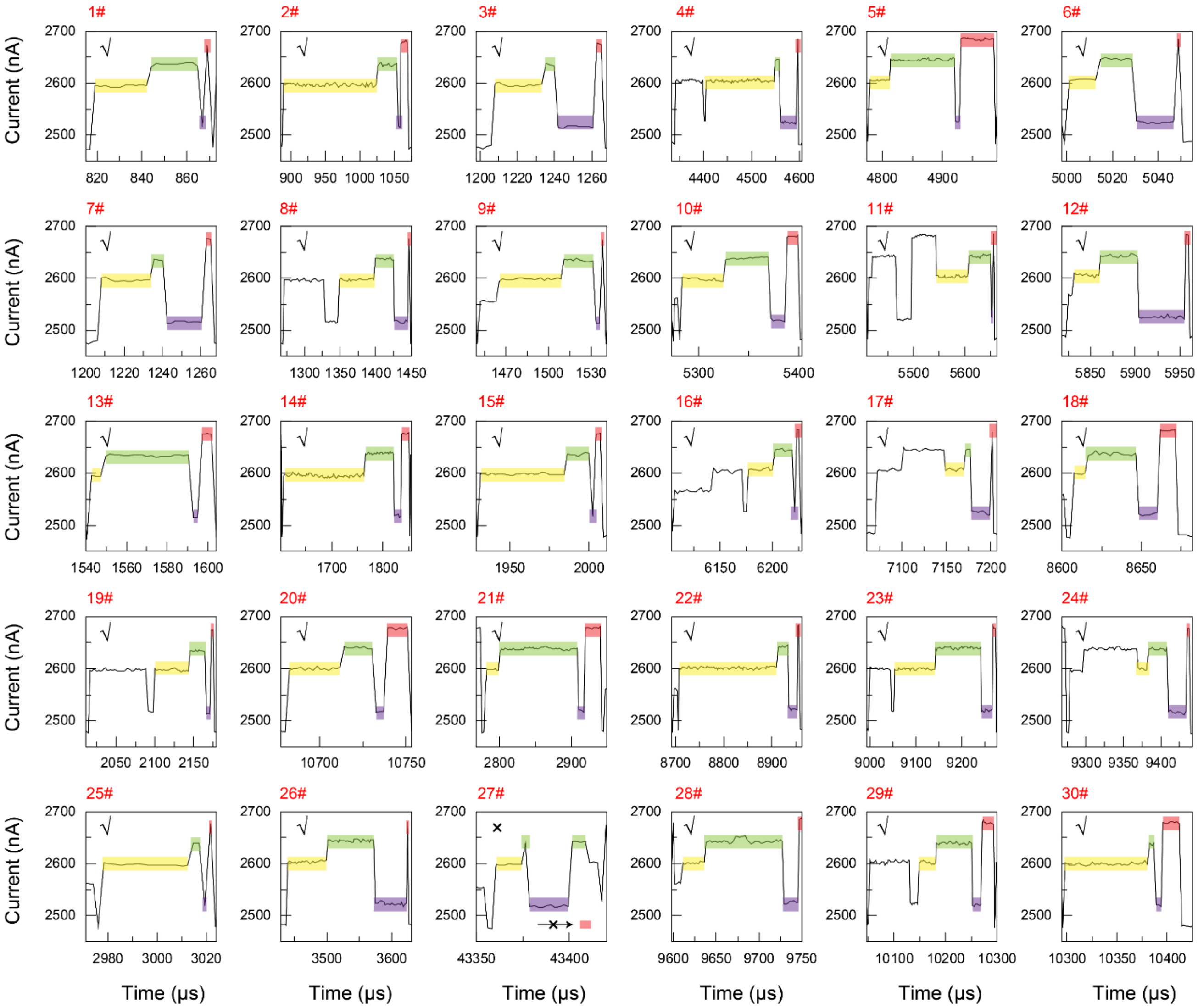

Figure S14. Representative acid-base processes monitored by *I*−*t* curves at 298 K and 1 V bias during ~10 ms. Most processes lead directly to the formation of products and return to the original catalyst state (Cat, marked as red). Only the process numbered 27 failed to successfully generate the product and returned to the (Cat-MA-CHO)H^+ state.

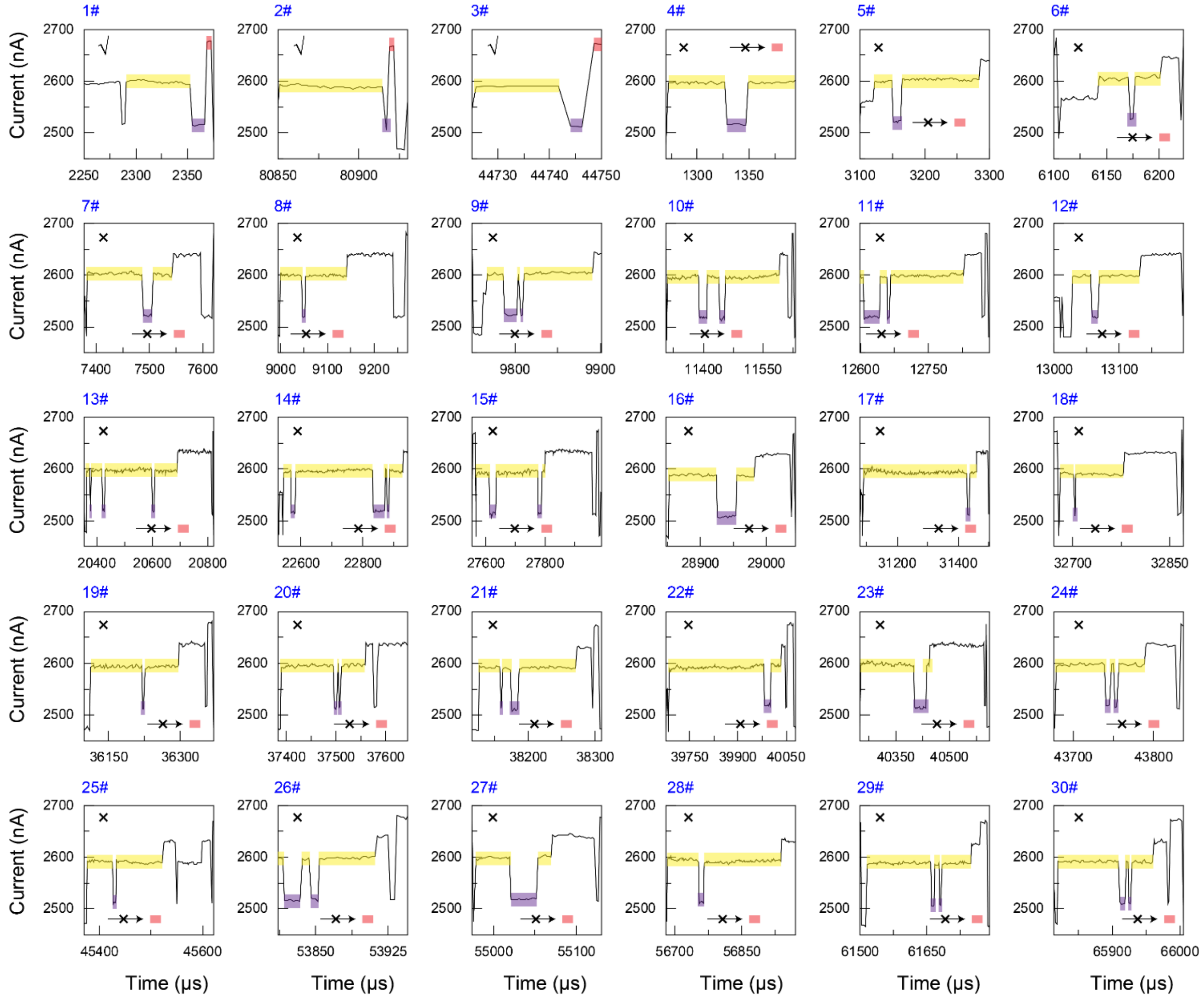

Figure S15. Representative proton shuttle processes monitored by *I*−*t* curves at 298 K and 1 V bias during ~66 ms. Most processes failed to successfully generate the product and returned to Cat-MA-CHO state. Only the processes numbered 1–3 lead directly to the formation of products and return to the original catalyst state (Cat, marked as red).

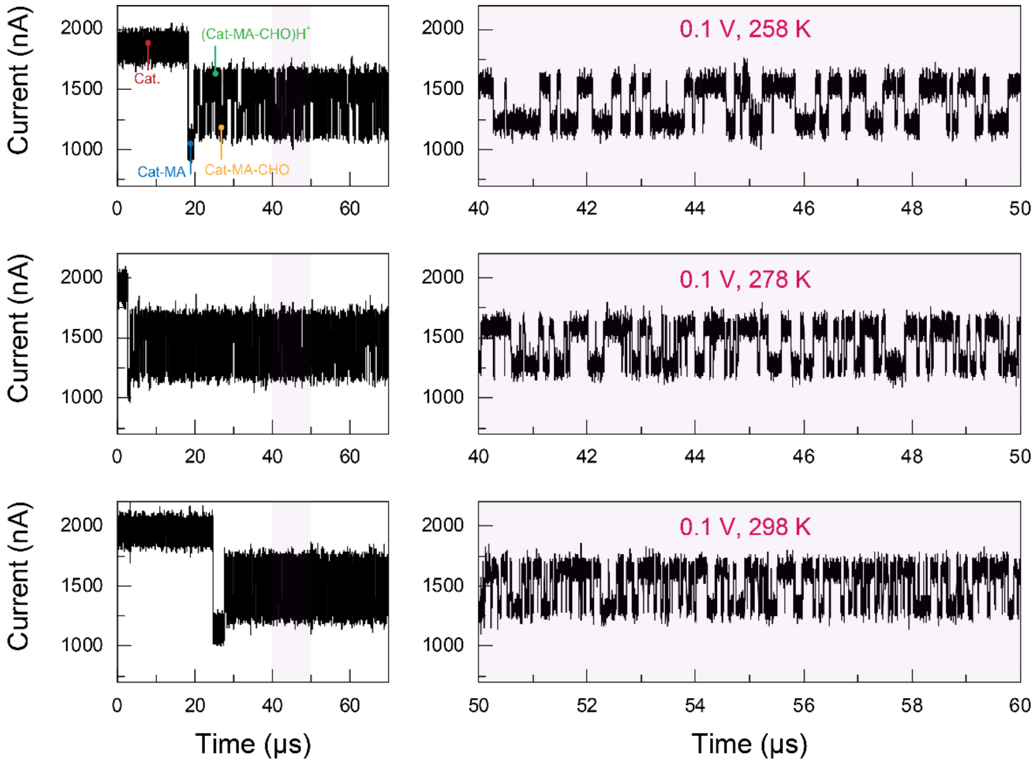

Figure S16. Temperature-dependent experiments of the proton shuttle at 0.1 V. In methanol solution, *I*−*t* curves for proton transfer at different temperatures are provided. The corresponding kinetic information in Fig. 4f is extracted here.

7. Characterization of the transesterification of the MBH reaction

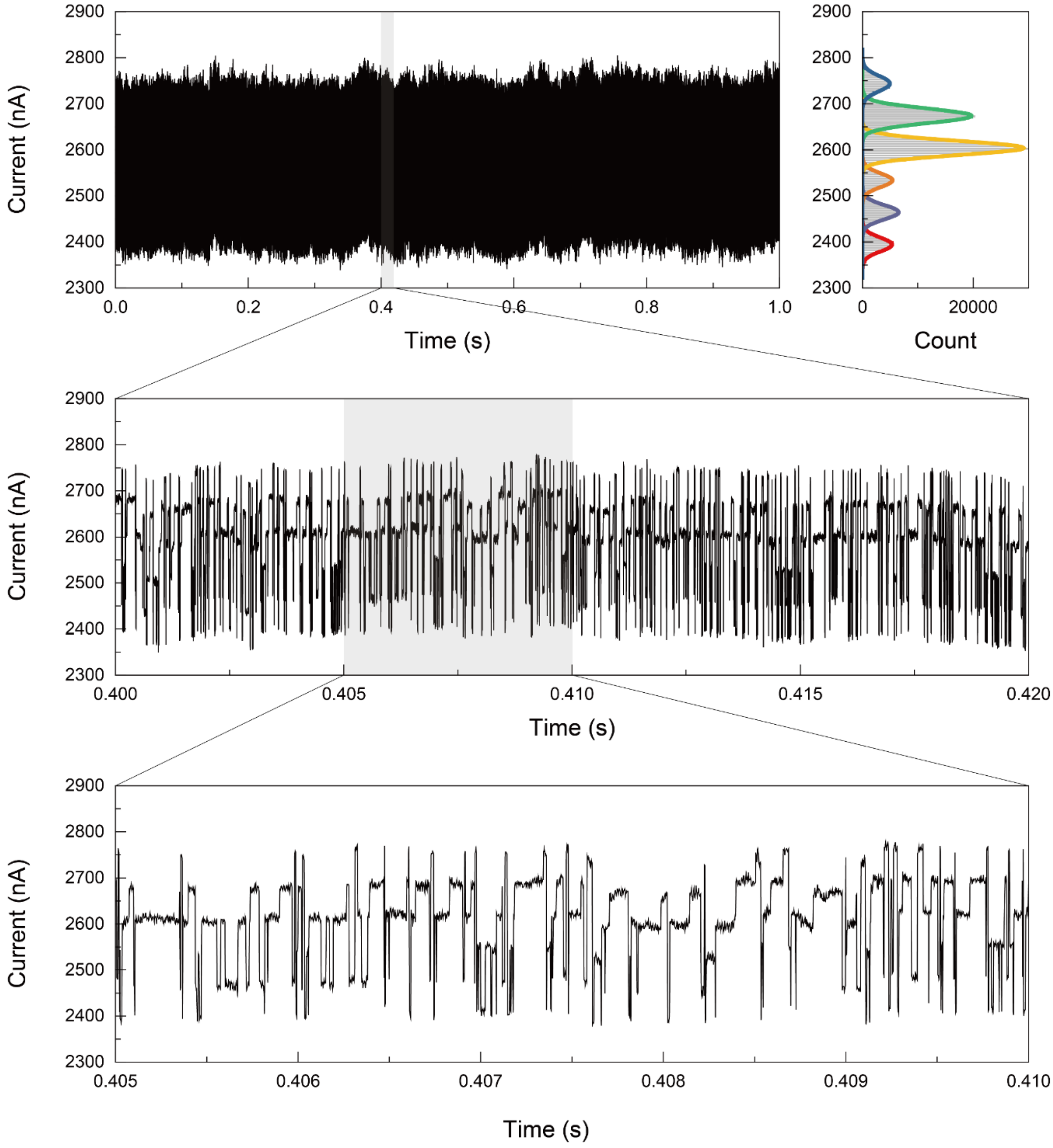

Figure S17. Electrical characterization of the MBH reaction using ethyl acrylate instead of methyl acrylate in the presence of methanol. The six Gaussian peaks in the histogram indicate that no transesterification occurred in the time range of 1 s. The enlarged images show the conductance switching time sequences, consistent with methyl acrylate as the substrate.

8. Reaction potential energy surfaces involving different proton transfer mechanisms

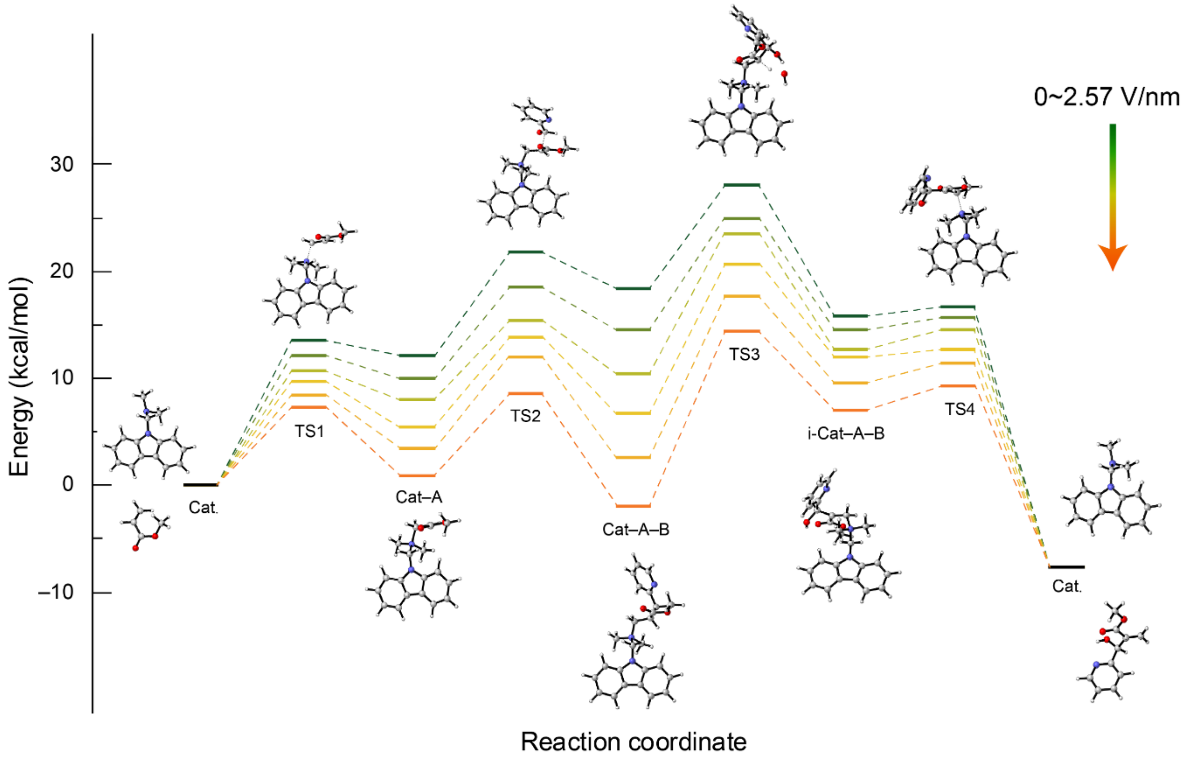

Figure S18. Simulated reaction potential energy surfaces of the MBH reaction with a proton transfer mechanism dominated by a proton shuttle process. Green to red indicates the presence of an external electrical field of 0 V/nm, 0.514 V/nm, 1.028 V/nm, 1.542 V/nm, 2.056 V/nm, and 2.57 V/nm, respectively. When an external electric field is applied along the molecular bridge, the component of dipole moments along the molecular bridge can be obviously strengthened, and the structures for the transition states can be energetically stabilized, leading to the reduction of energy barriers.

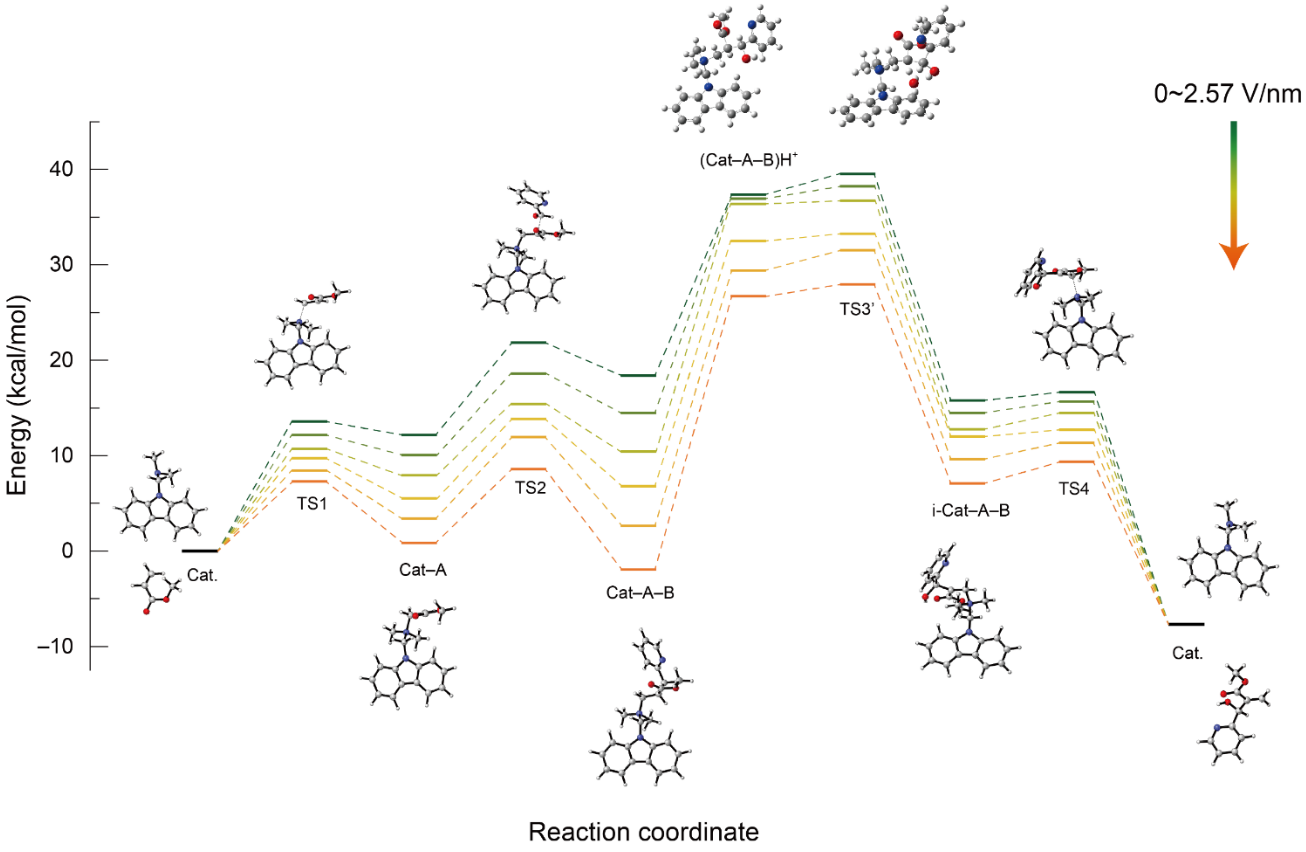

Figure S19. Simulated reaction potential energy surfaces of the MBH reaction with a proton transfer mechanism dominated by an acid-base process. Green to red indicates the presence of an external electrical field of 0 V/nm, 0.514 V/nm, 1.028 V/nm, 1.542 V/nm, 2.056 V/nm, and 2.57 V/nm, respectively.

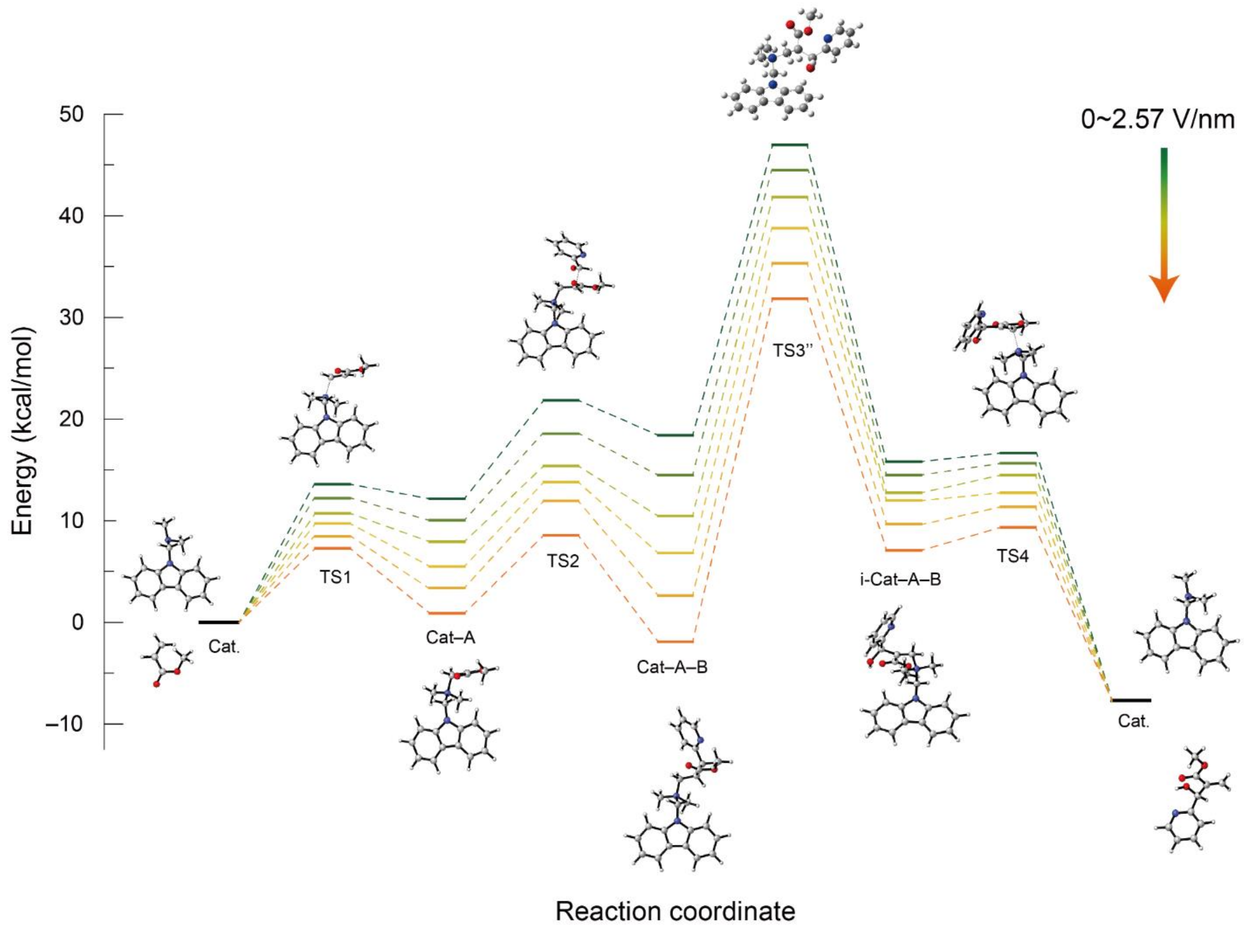

Figure S20. Simulated reaction potential energy surfaces of the MBH reaction with a proton transfer mechanism dominated by a four-member ring transition state. Green to red indicates the presence of an external electrical field of 0 V/nm, 0.514 V/nm, 1.028 V/nm, 1.542 V/nm, 2.056 V/nm, and 2.57 V/nm, respectively.

Table S1. The enthalpy of the first two steps calculated under different electric fields.

	**0 V/nm**	**0.514 V/nm**	**1.028 V/nm**	**1.542 V/nm**	**2.056 V/nm**	**2.57 V/nm**
**Cat**	0.00	0.00	0.00	0.00	0.00	0.00
**TS1**	−1.04	−2.30	−3.56	−4.76	−5.97	−7.25
**Cat-MA**	−2.71	−4.76	−6.90	−9.18	−11.60	−14.14
**TS2**	−7.45	−10.41	−13.22	−14.32	−16.53	−19.15
**Cat-MA-CHO**	−11.16	−14.96	−18.89	−22.97	−27.24	−31.71

9. Characterization of the single-molecule catalytic oscillations

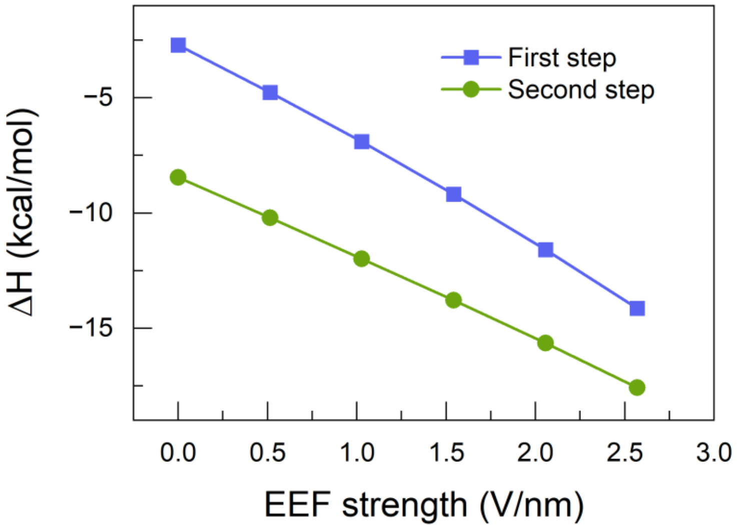

Figure S21. The relationship between the enthalpy changes and the electric field strength was obtained by computational simulations. It can be found that the exothermic heat increases with the increase of an electric field.

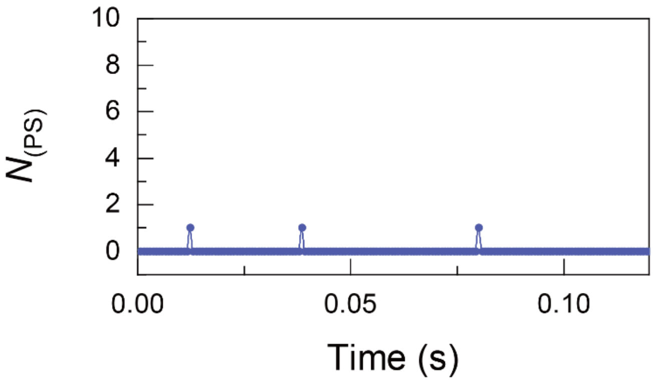

Figure S22. The control experiment of the single-molecule chemical oscillation in a flowing solution. The number of molecules of the product produced was recorded at 0.7 ms intervals in the flowing solution (ultradry DMSO, rate: 1 mL/min) at 298 K. No periodic formation of products was observed, excluding the MBH reaction mechanism dominated by the four-number ring transition state.

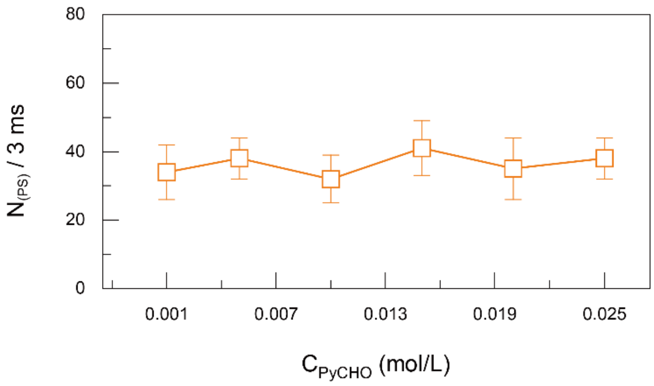

Figure S23. Effect of aldehyde concentration on proton transfer. At 298 K, with the addition of different concentrations of pyridine carboxaldehyde, the number of products was recorded every 3 ms. Aldehydes have no significant effect on the rate of product formation, indicating that aldehyde-assisted proton transfer is not dominant.

10. Bias dependence of the whole MBH reaction

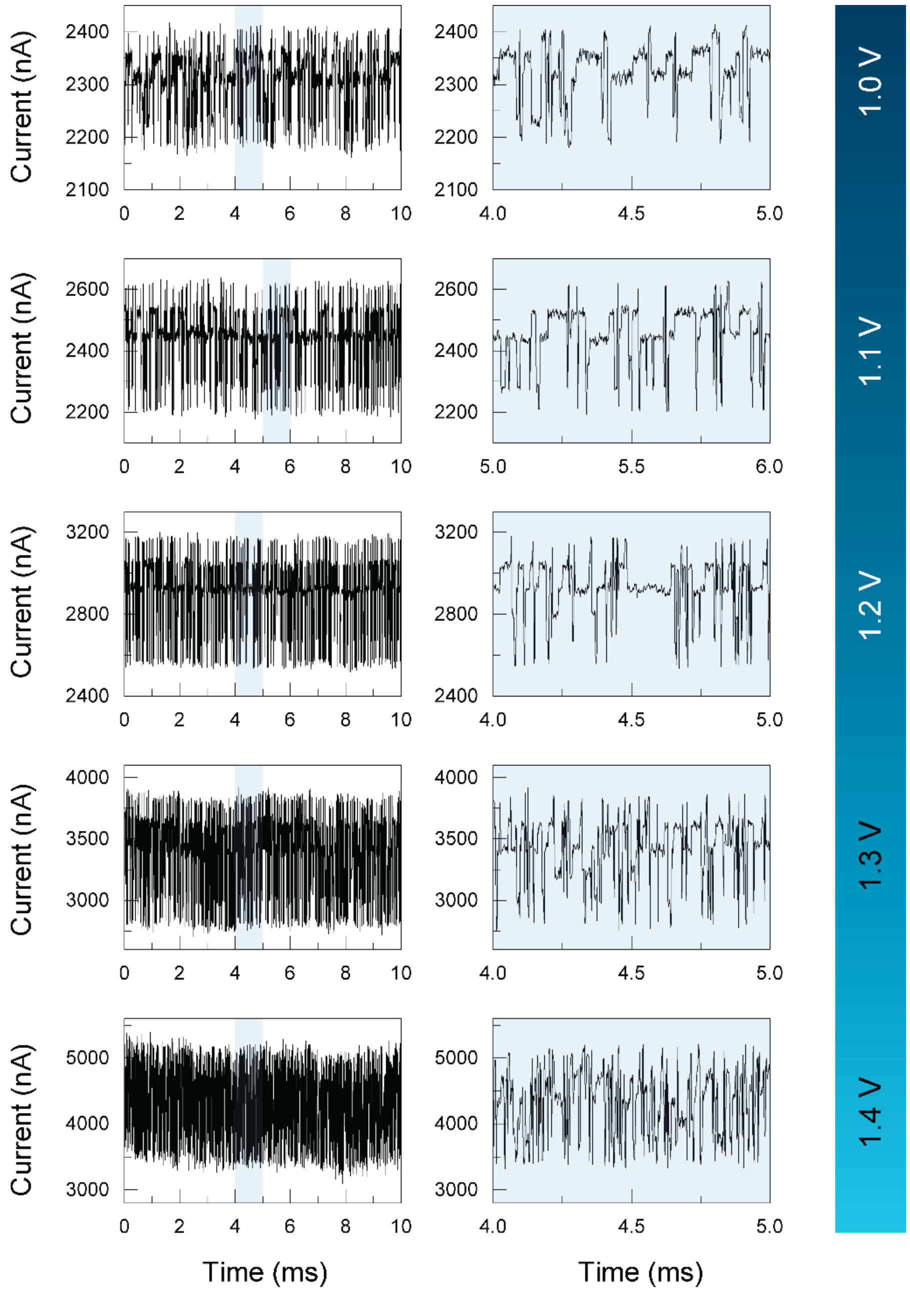

Figure S24. Bias voltage-dependent experiments of the MBH reaction at 298 K. *I*–*t* curves and enlarged images of the single-molecule device at different bias voltages with adding a MeOH solution of 10^{-3} mol/L MA and 10^{-3} mol/L PyCHO. With the increase of bias voltage, the period of one catalysis cycle decreased, indicating that the electric field effectively drove all steps.

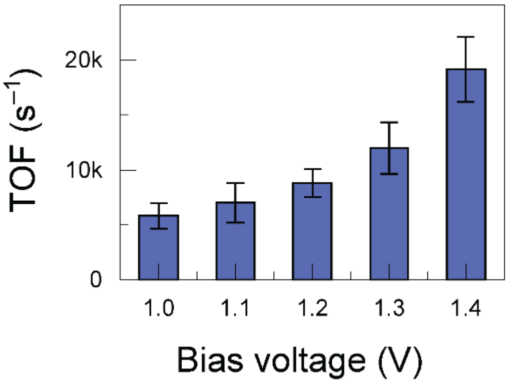

Figure S25. Statistics to the TOF of the MBH reaction catalyzed by EEF. The experiment was conducted at different bias voltages with adding a MeOH solution of 10^{-3} mol/L MA and 10^{-3} mol/L PyCHO. The corresponding TOFs were obtained by counting the catalysis cycles.

11. Characterization of the macroscale synthesis

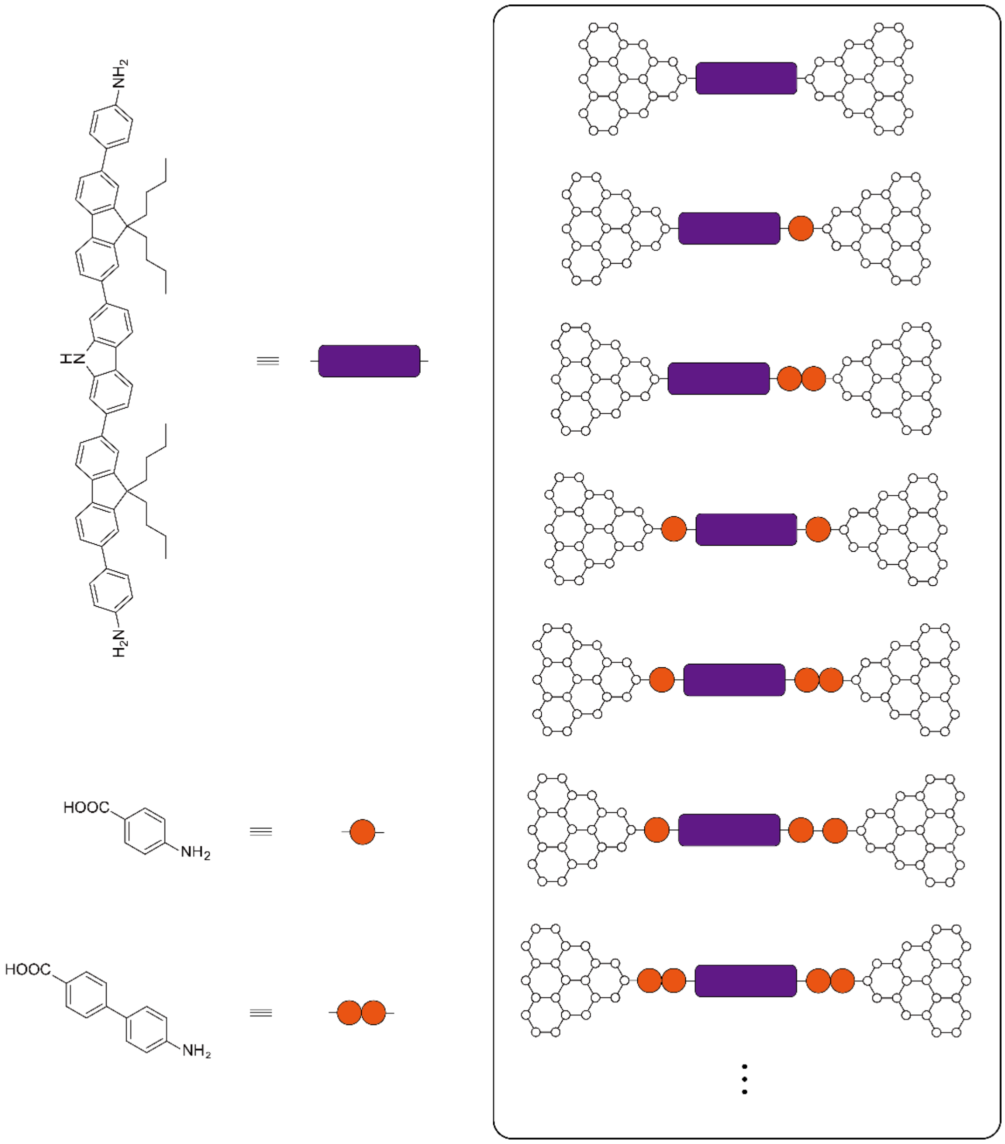

Figure S26. Strategy to integrate multiple single-molecule catalysts, showing two kinds of bridge piers and how to integrate multiple molecules with different lengths into graphene nanogaps with a range of widths (1~10 nm).

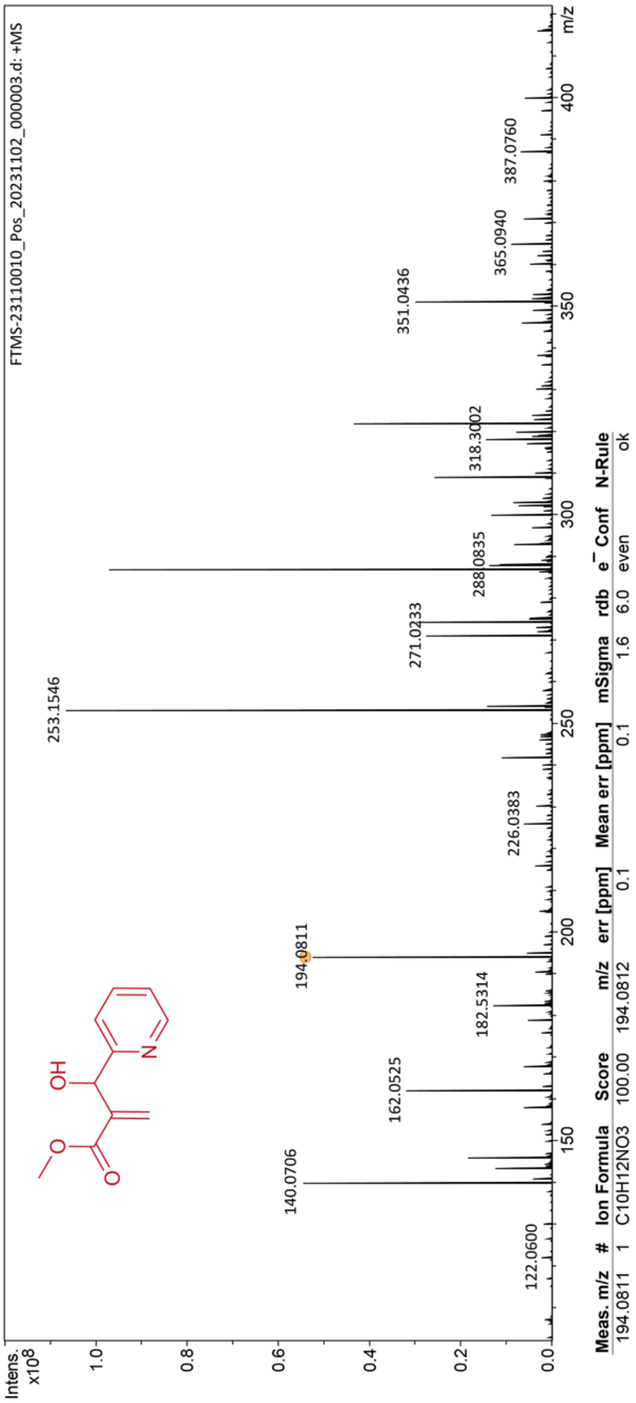

Figure S27. Macroscale synthesis of methyl 2-(hydroxy(pyridin-2-yl)methyl)acrylate by one individual single-catalyst device at 298 K and 1 V bias voltage. The single-molecule catalysis took two months to reach macroscopically detectable standards (by high-resolution mass spectrometry (HRMS)).

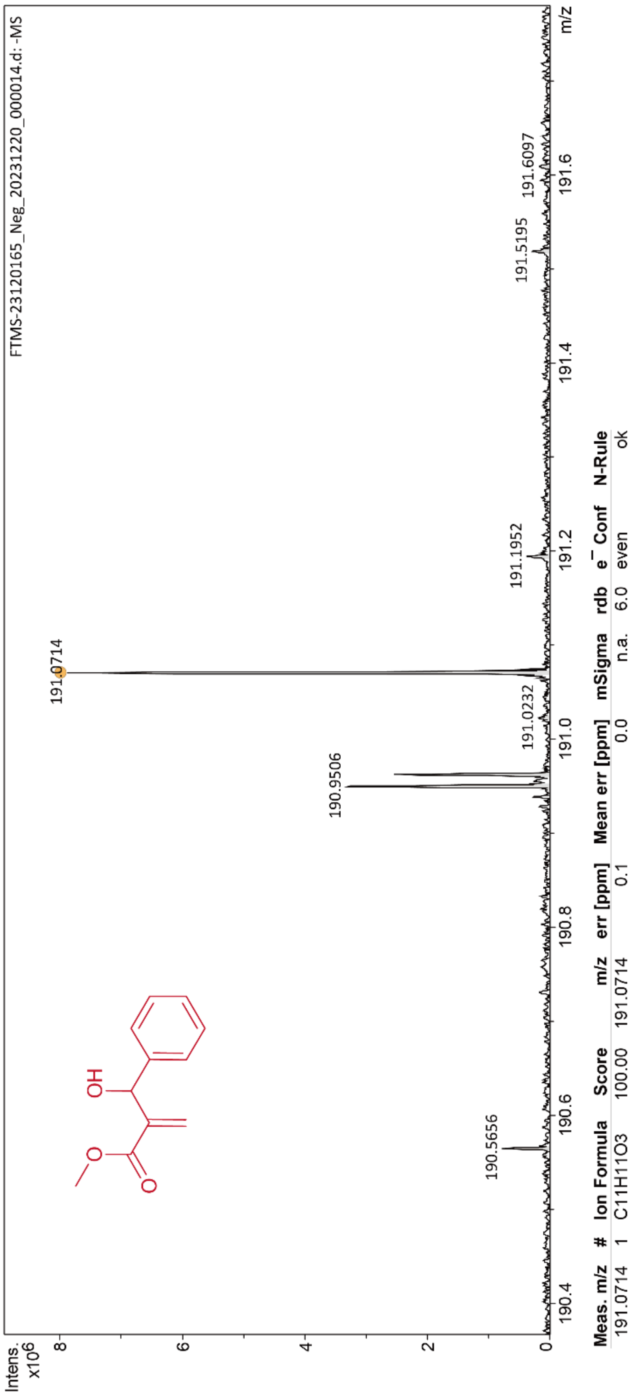

Figure S28. Macroscale synthesis of methyl 2-(hydroxy(phenyl)methyl)acrylate by a single-catalyst array on chip at 298 K and 1 V bias voltage. The single-molecule catalysis took 1 h to reach macroscopically detectable standards and characterized by HRMS.

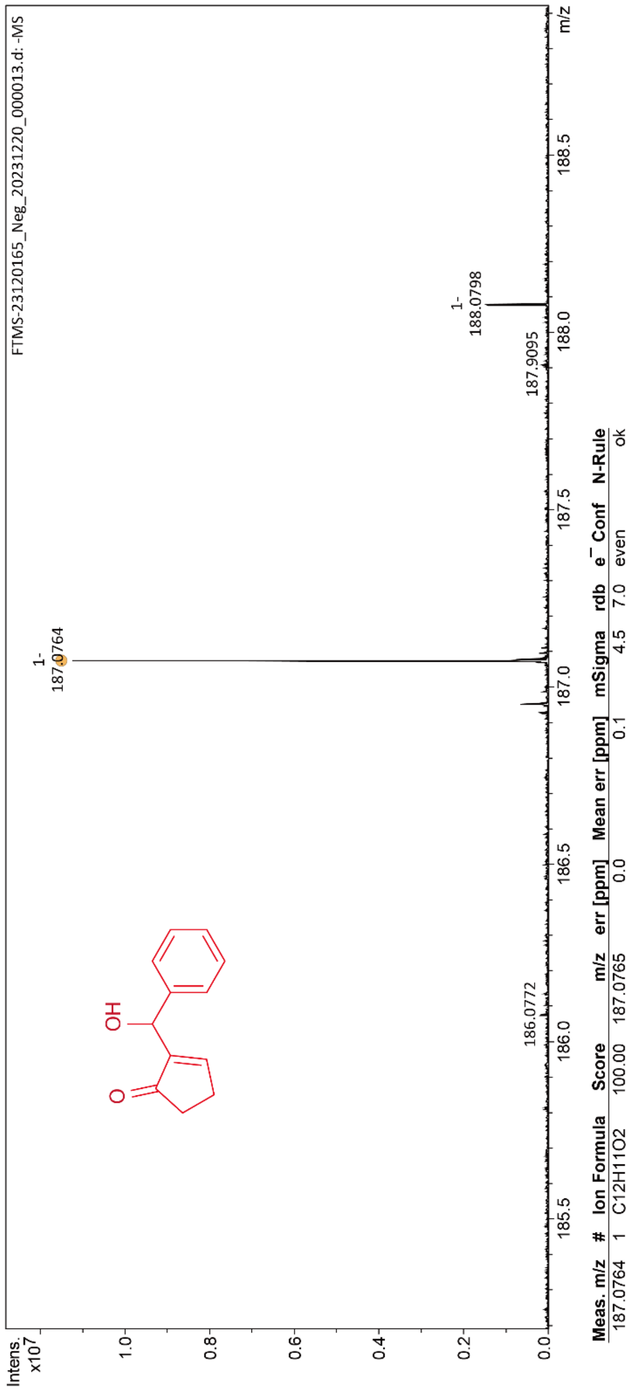

Figure S29. Macroscale synthesis of 2-(hydroxy(phenyl)methyl)cyclopent-2-en-1-one by a single-catalyst array on chip at 298 K and 1 V bias voltage. The single-molecule catalysis took 1 h to reach macroscopically detectable standards and characterized by HRMS.

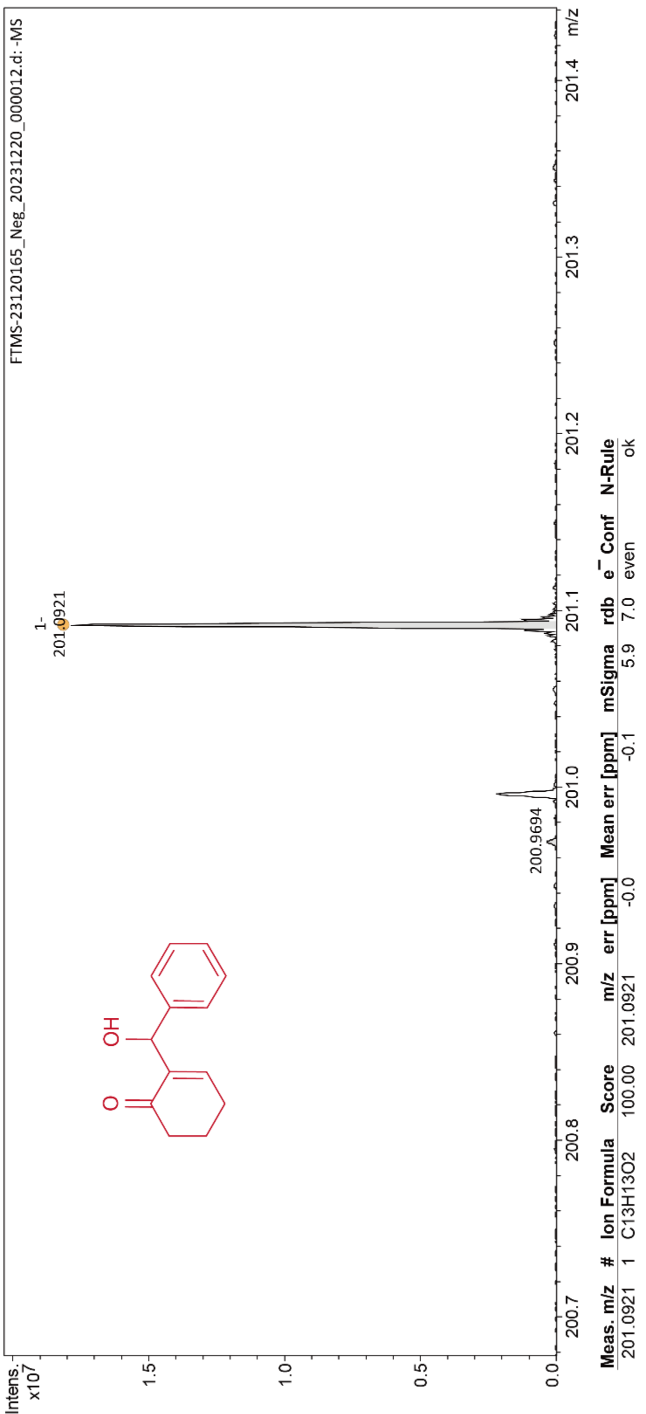

Figure S30. Macroscale synthesis of 2-(hydroxy(phenyl)methyl)cyclohex-2-en-1-one by a single-catalyst array on chip at 298 K and 1 V bias voltage. The single-molecule catalysis took 1 h to reach macroscopically detectable standards and characterized by HRMS.

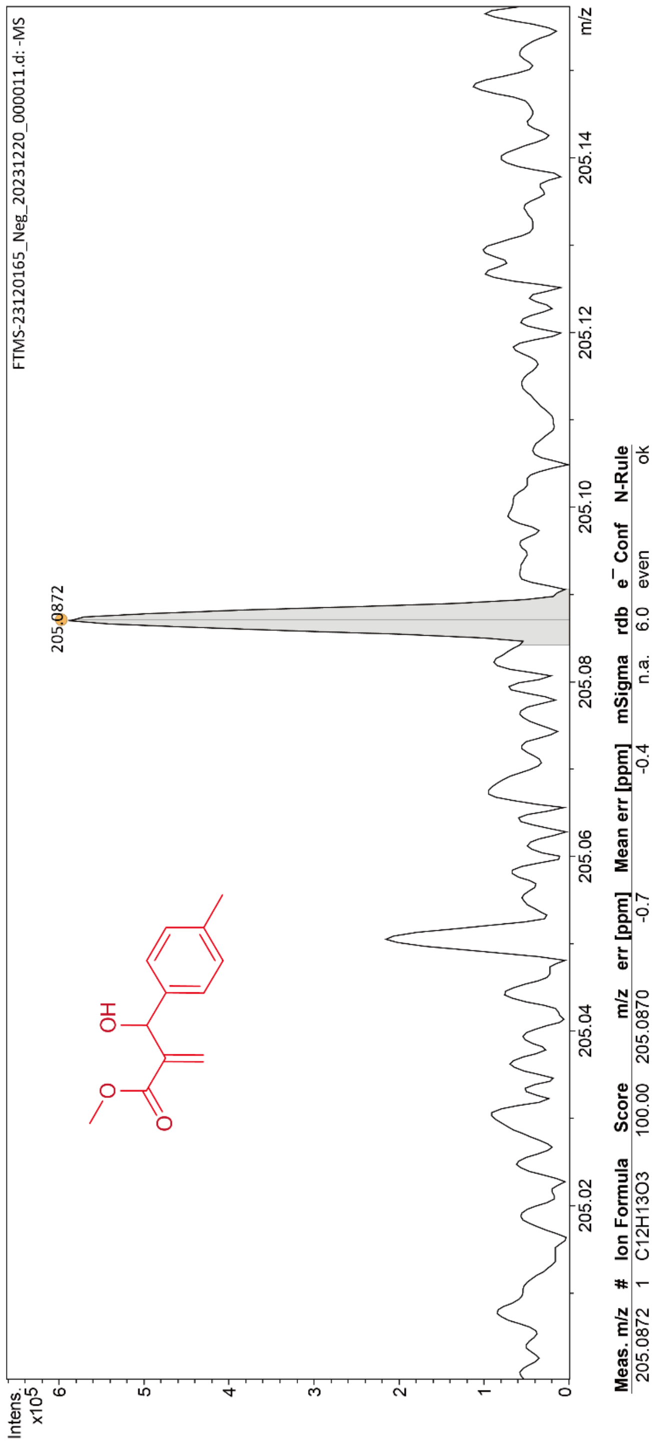

Figure S31. Macroscale synthesis of methyl 2-(hydroxy(p-tolyl)methyl)acrylate by a single-catalyst array on chip at 298 K and 1 V bias voltage. The single-molecule catalysis took 1 h to reach macroscopically detectable standards and characterized by HRMS.

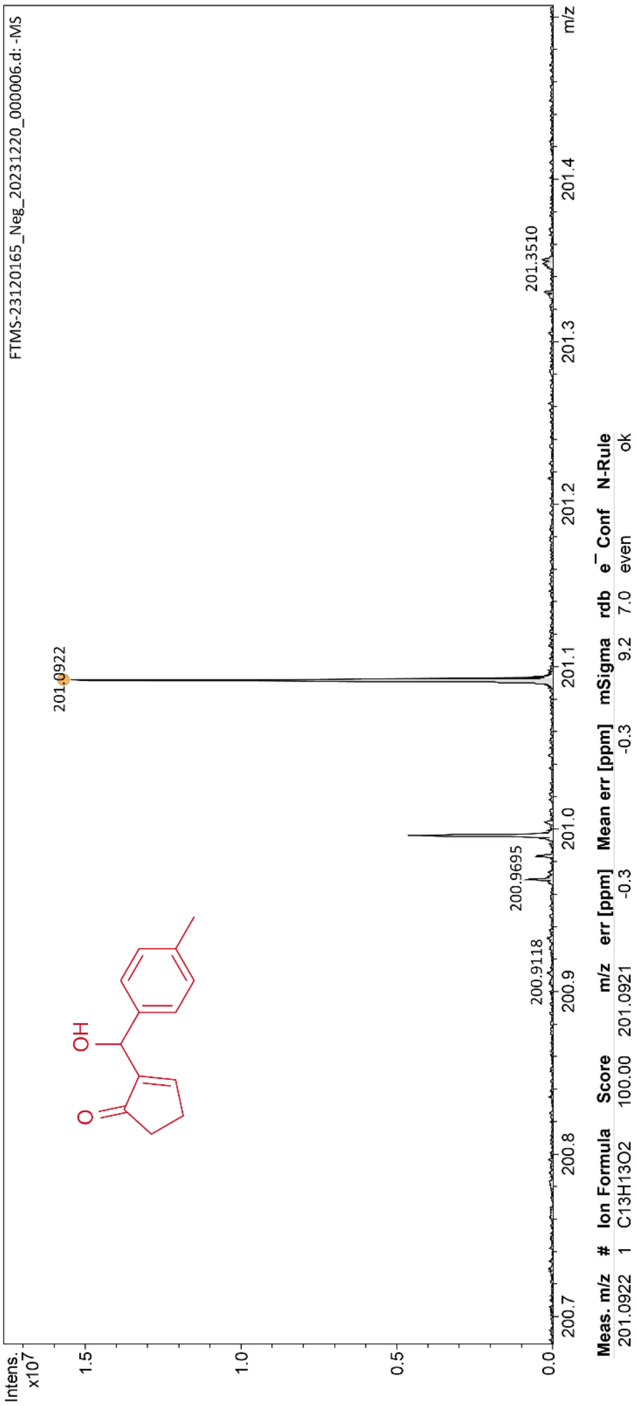

Figure S32. Macroscale synthesis of 2-(hydroxy(p-tolyl)methyl)cyclopent-2-en-1-one by a single-catalyst array on chip at 298 K and 1 V bias voltage. The single-molecule catalysis took 1 h to reach macroscopically detectable standards and characterized by HRMS.

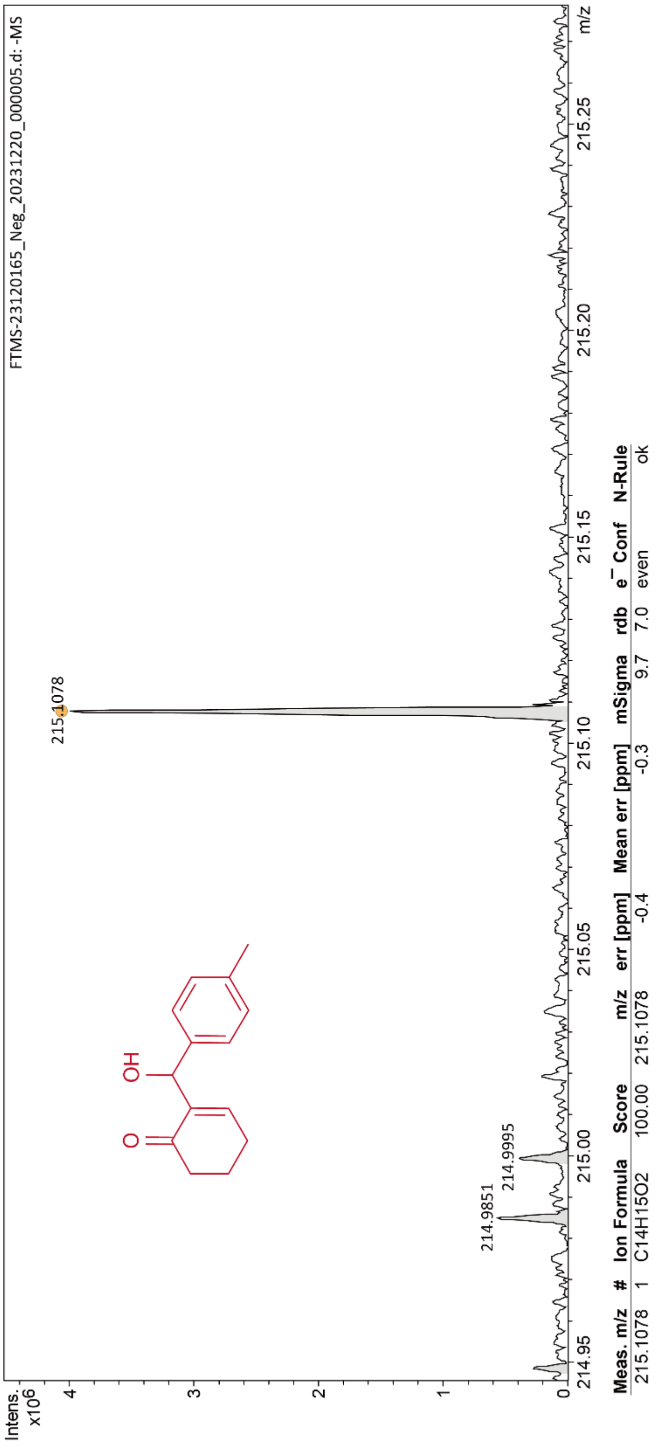

Figure S33. Macroscale synthesis of 2-(hydroxy(p-tolyl)methyl)cyclohex-2-en-1-one by a single-catalyst array on chip at 298 K and 1 V bias voltage. The single-molecule catalysis took 1 h to reach macroscopically detectable standards and characterized by HRMS.

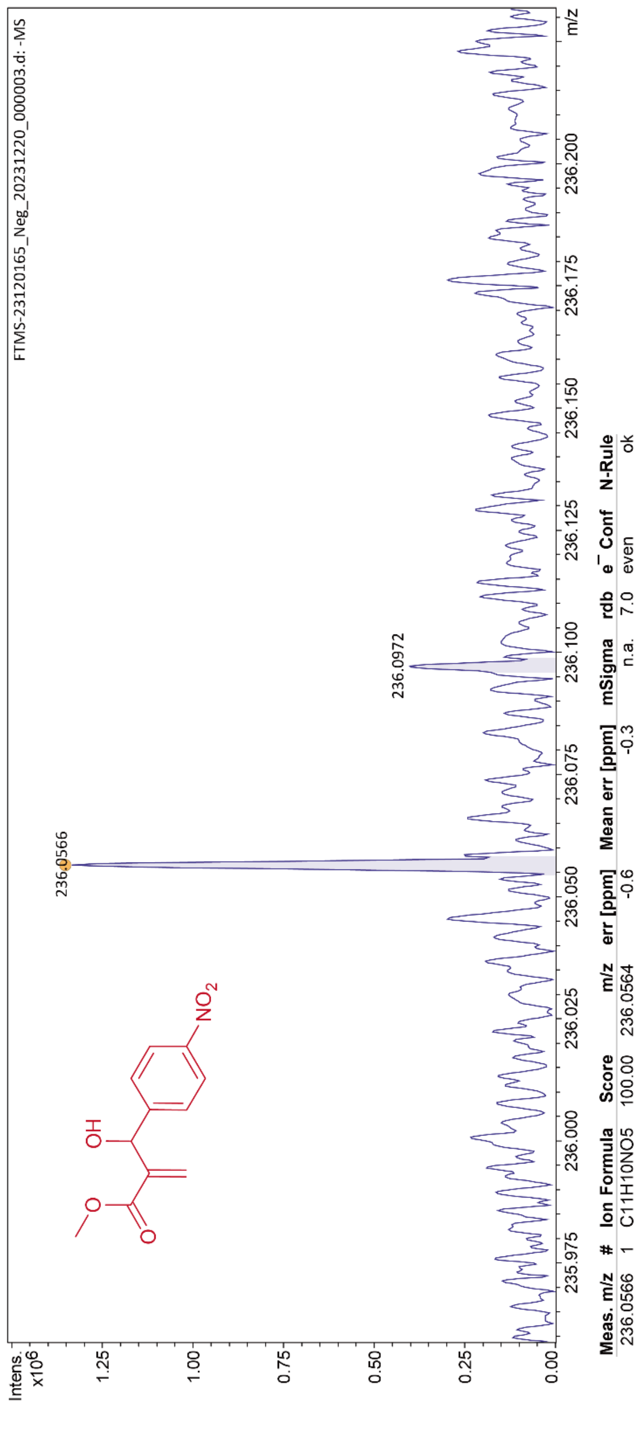

Figure S34. Macroscale synthesis of methyl 2-(hydroxy(4-nitrophenyl)methyl)acrylate by a single-catalyst array on chip at 298 K and 1 V bias voltage. The single-molecule catalysis took 1 h to reach macroscopically detectable standards and characterized by HRMS.

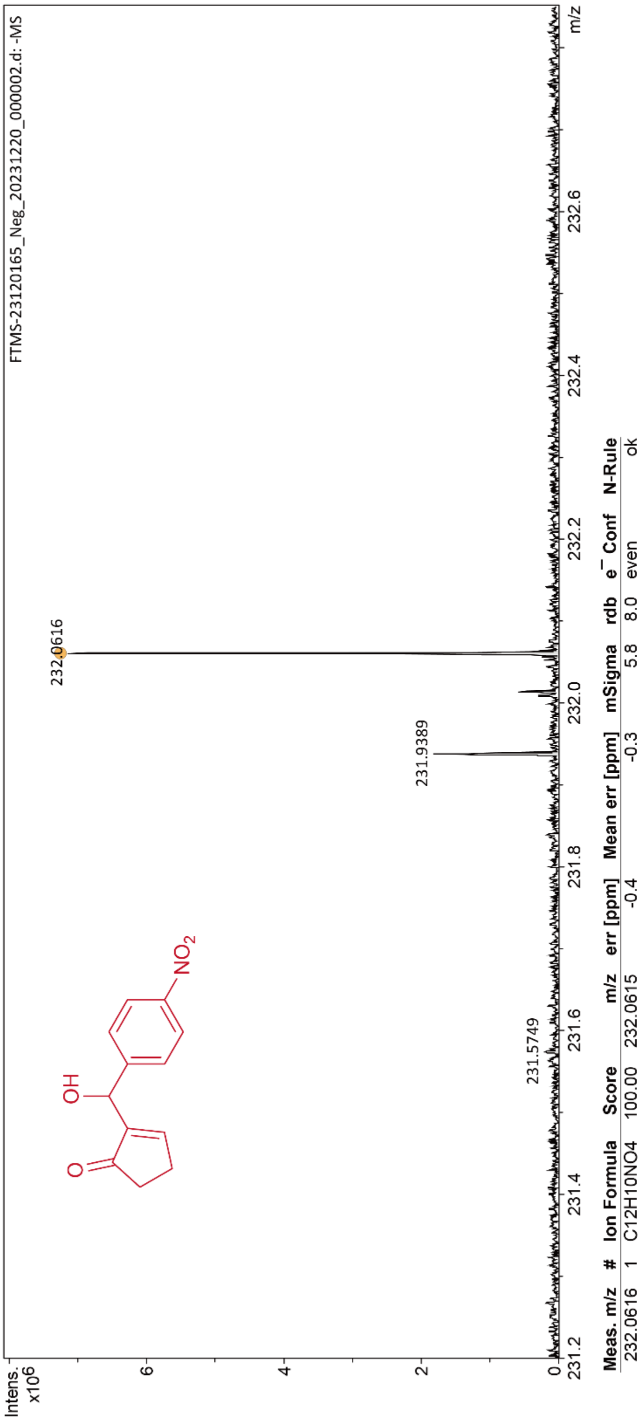

Figure S35. Macroscale synthesis of 2-(hydroxy(4-nitrophenyl)methyl)cyclopent-2-en-1-one by a single-catalyst array on chip at 298 K and 1 V bias voltage. The single-molecule catalysis took 1 h to reach macroscopically detectable standards and characterized by HRMS.

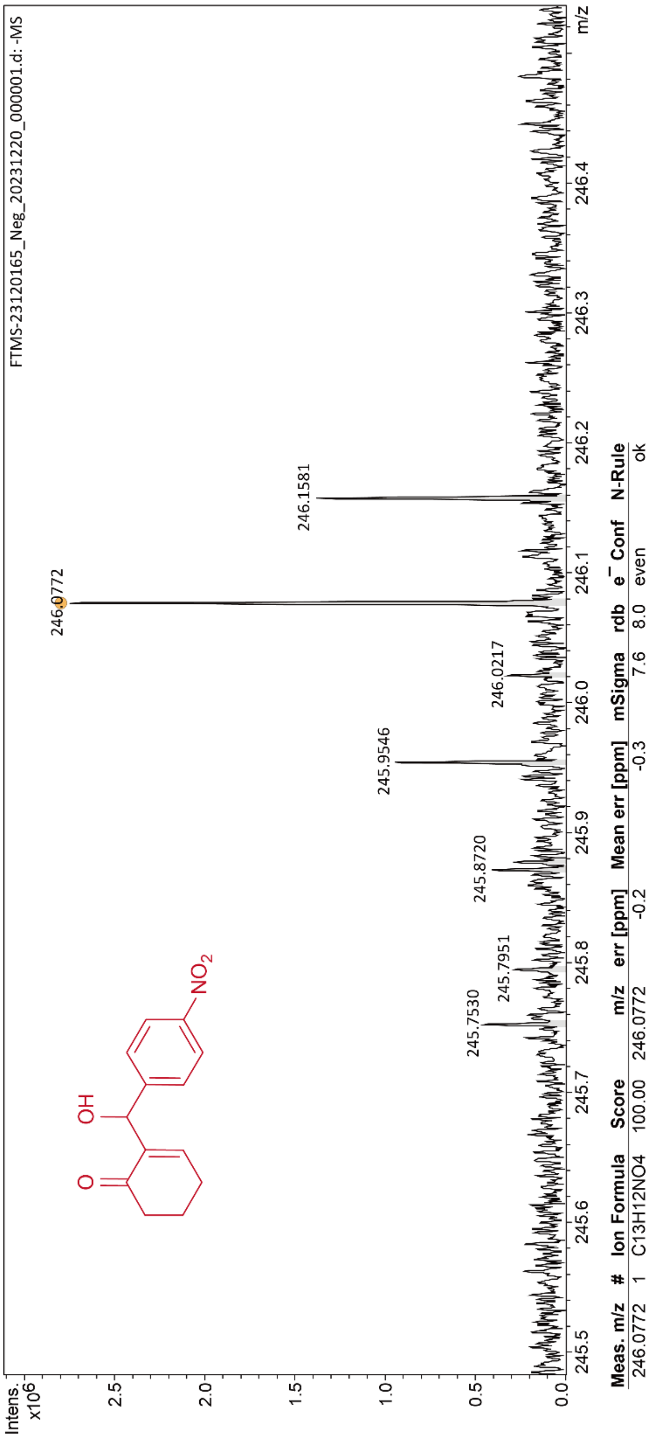

Figure S36. Macroscale synthesis of 2-(hydroxy(4-nitrophenyl)methyl)cyclohex-2-en-1-one by a single-catalyst array on chip at 298 K and 1 V bias voltage. The single-molecule catalysis took 1 h to reach macroscopically detectable standards and characterized by HRMS.

References

1 Kawano S, Kato M, Soumiya S *et al.* Columnar liquid crystals from a giant macrocycle mesogen. *Angew Chem Int Ed* 2018; **57**: 167–71.

2 Guo Y, Yang C, Li H *et al.* Accurate single-molecule kinetic isotope effects. *J Am Chem Soc* 2022; **144**: 3146–53.

3 Frisch M, Schlegel HB, Scuseria GE *et al.* Gaussian 09, Revision D. 01. *Gaussian Inc.: Wallingford, CT*; 2013.

4 Zhao Y, Truhlar DG. The M06 suite of density functionals for main group thermochemistry, thermochemical kinetics, noncovalent interactions, excited states, and transition elements: two new functionals and systematic testing of four M06-class functionals and 12 other functionals. *Theor Chem Acc* 2008; **120**: 215−41.

5 Marenich AV, Cramer CJ, Truhlar DG. Universal solvation model based on solute electron density and on a continuum model of the solvent defined by the bulk dielectric constant and atomic surface tensions. *J Phys Chem B* 2009; **113**: 6378−96.

6 Lu T, Chen Q. Shermo: A general code for calculating molecular thermochemistry properties. *Comput Theor Chem* 2021; **1200**: 113249.

7 Rex S, Donald T, Bruce G, A general small-curvature approximation for transition-state-theory transmission coefficients. *J Phys Chem* 1981; **85**: 3019−23.

8 Doubleday C, Armas R, Walker D, Cosgriff V, Greer M, Heavy-atom tunneling calculations in thirteen organic reactions: Tunneling contributions are substantial, and bell's formula closely approximates multidimensional tunneling at ⩾250 K. *Angew. Chem. Int. Ed.* 2017; **56**: 13099.

9 Smidstrup S, Markussen T, Vancraeyveld P *et al.* QuantumATK: an integrated platform of electronic and atomic-scale modelling tools. *J Phys Condens Matter* 2020; **32**: 015901.
